# Supplementary figures and images for: Distinct structural groups of histone H3 and H4 residues have divergent effects on chronological lifespan in Saccharomyces cerevisiae
Source: PLoS One. 2022 May 27;17(5):e0268760. doi: 10.1371/journal.pone.0268760 (PMC9140238; doi:10.1371/journal.pone.0268760)

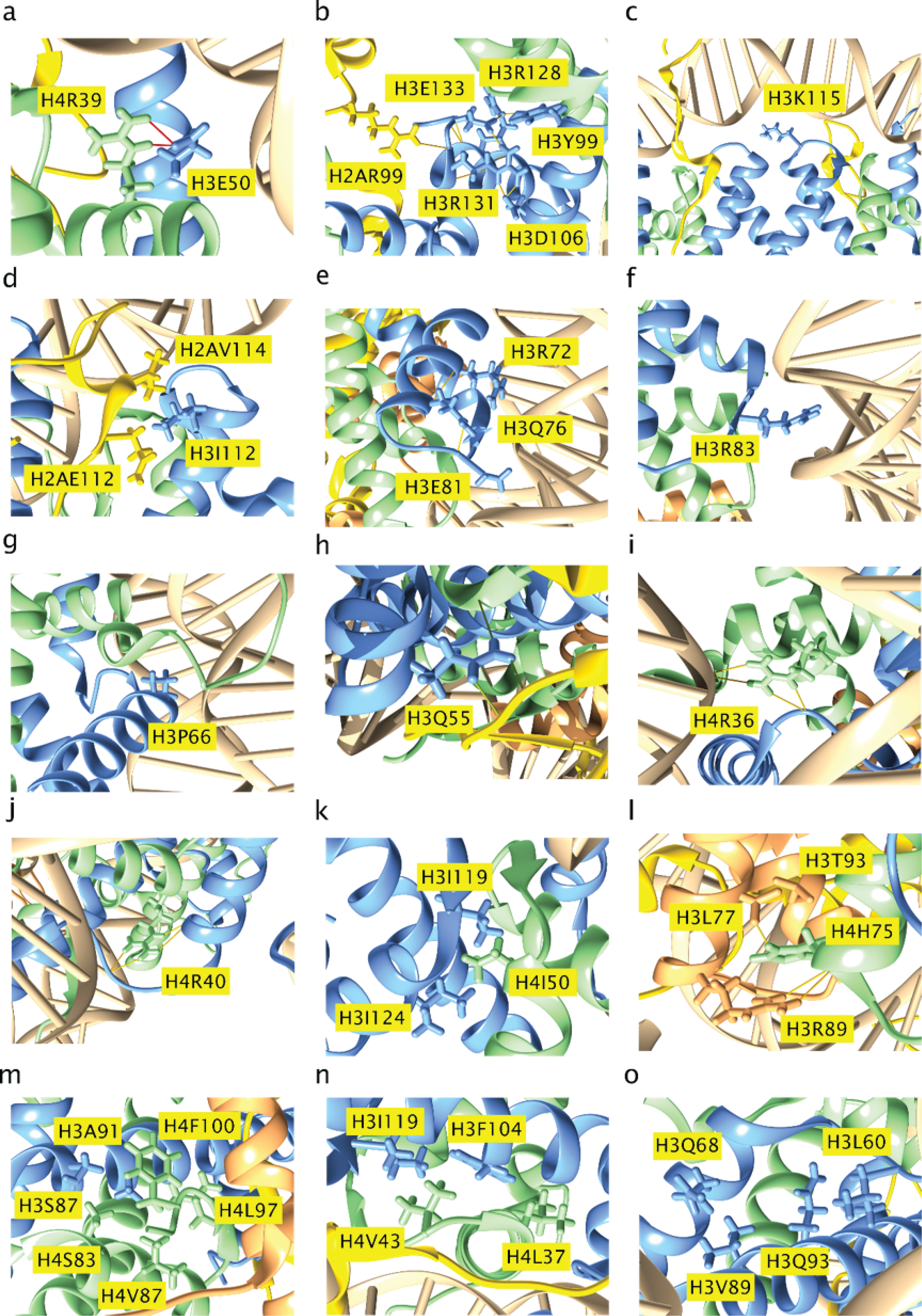

Supplement: S1 Fig — H3E50 makes hydrogen bonds to H4R39 (a). H3R128 and H3R131 make H-bonds to H3Y99 and H3D106 in α2, and H3R131 makes an H-bond to R99 in the Cα helix of H2A (b). H3K115 is oriented towards a phosphate group in the DNA sugar-phosphate backbone (c). H3I112 forms a hydrophobic pocket with H2AQ112 and H2AV114 (d). H3Q76 makes bridging H-bonds to the backbone at H3D81 in L1 and H3R72 in α1 (e). R83 of H3 is inserted into the minor groove at SHL ±2.5 (f). H3P66 at the N-terminus of α1 may contribute to the termination the α1 helix (g). H3Q55 is hydrogen bonded to the H2A C-terminal tail backbone at N110 as well as to R40 in α1 of H4 (h). H4R36 make salt bridge contacts with a phosphate group in the DNA sugar-phosphate backbone (i). H4R40 makes H-bonds to the H3 backbone αN and αN-α1 connecting coil (j). H4I50 is inserted between H3I119 and H3I124 (k). H4H75 in α2 of H4 make hydrophobic contact with H2BL77 and H2BT93, as well as an H-bond to H2BR89 (l). H4F100 makes numerous hydrophobic contacts with H3S87, H3A91, H4A83, H4V87 and H4L97 (m). H3F104 is inserted into a hydrophobic cluster formed by H3I119, H4L37 and H4V43 (n). Hydrophobic contacts between H3L60 and H3Q93 and between H3Q68 and H3V89 stabilizes the conformation of H3 α1 relative to H3 α2 (o). (TIF) [file pone.0268760.s001.tif]

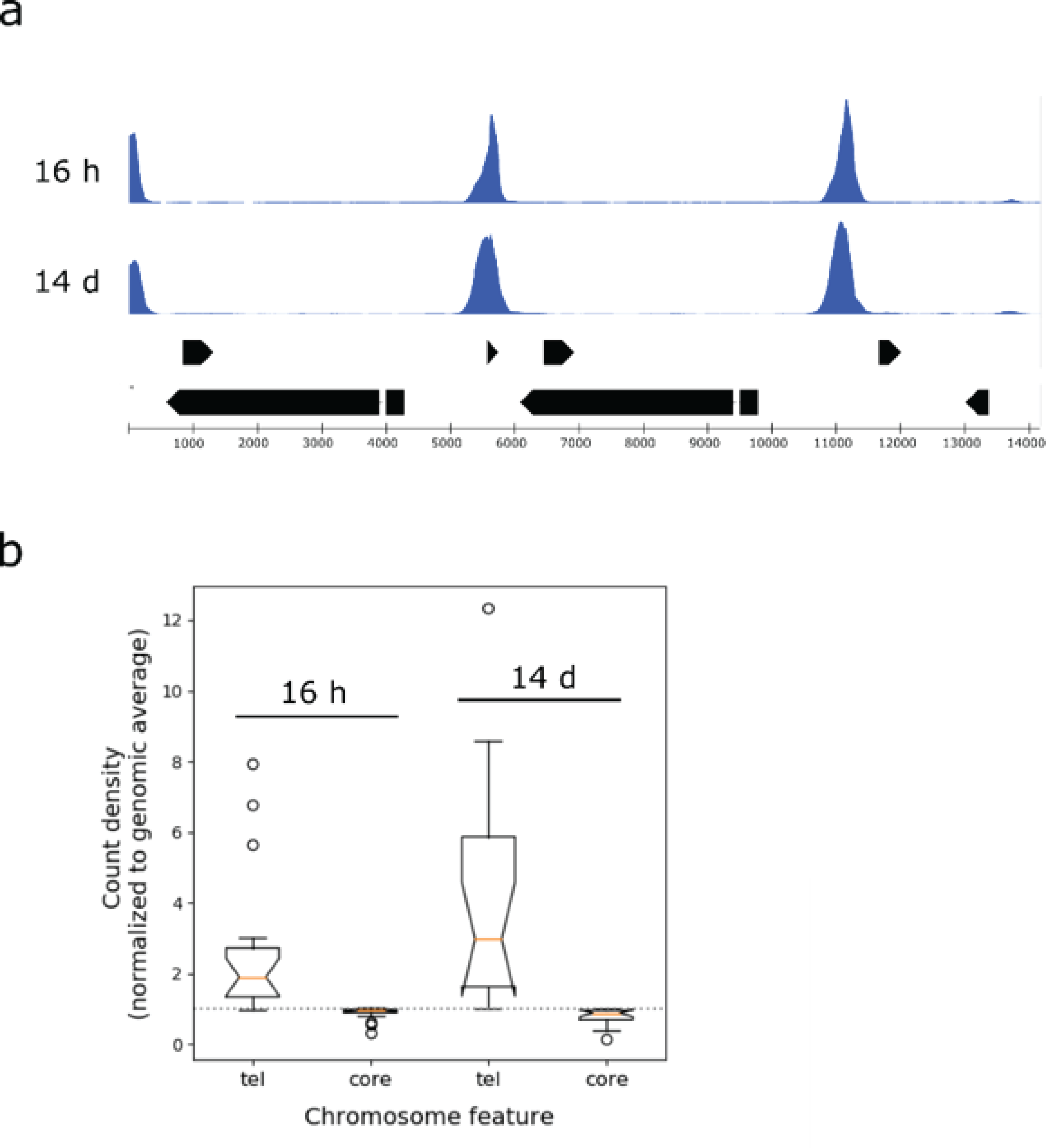

Supplement: S2 Fig — The normalised binding of Rap 1 is shown at 16 h and 14 d for the left telomeric 15 kb of chromosome XII (a) and to the terminal 20 kb (tel) or remaining central region (core) of all 16 chromosomes normalised to the genome average (b). The box plots represent the inter-quartile range (IQR), the notch represents the significance at p<0.05, the orange line shows the median, and the whiskers is shown at 1.5× the IQR. Outliers are shown as individual data points. There is no significant difference (p<0.05, t-test) between the corresponding data groups. (TIF) [file pone.0268760.s002.tif]

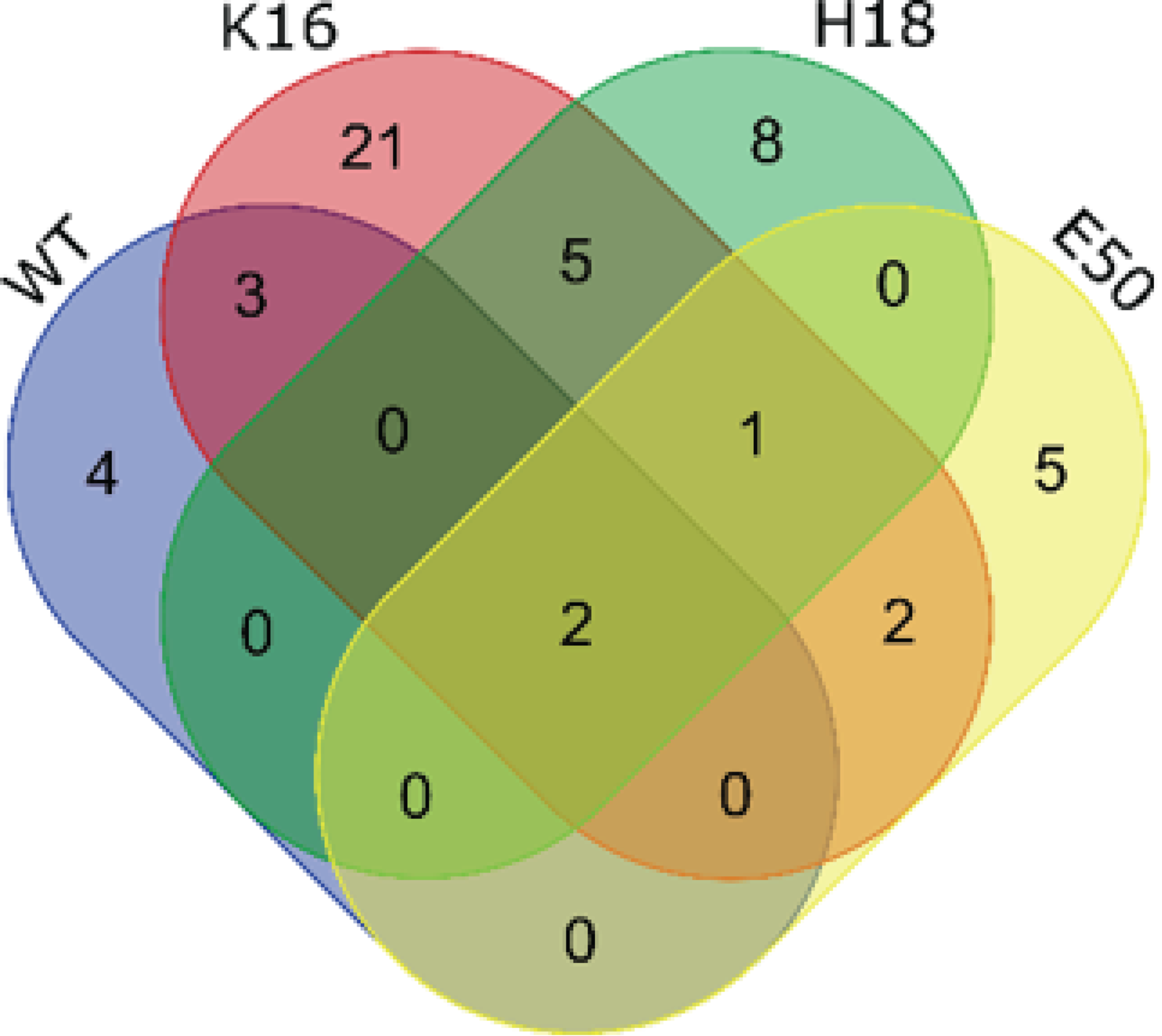

Supplement: S3 Fig — The Venn diagram shows the number of genes associated with Sir3 peaks that are common to various combinations of the H4K16Q, H4H18A, H3E50A and WT strains. (TIF) [file pone.0268760.s003.tif]
