# Supplementary material for: Distinct structural groups of histone H3 and H4 residues have divergent effects on chronological lifespan in Saccharomyces cerevisiae
Source: PLoS One. 2022 May 27;17(5):e0268760. doi: 10.1371/journal.pone.0268760 (PMC9140238; doi:10.1371/journal.pone.0268760)
Supplement: S1 Table — The ratio of the barcode associated with each different strain was normalised to that of the same barcode at time t0, and expressed as log2. The rows are sorted in descending order on the 44 d column. (DOCX) [file pone.0268760.s004.docx]

| **S1 Table. The viability of different histone mutant strain in stationary phase.** The ratio of the barcode  associated with each different strain was normalised to that of the same barcode at time t0, and expressed as log_2_.  The rows are sorted in descending order on the 44 d column. | | | | | | | | | | |
| --- | --- | --- | --- | --- | --- | --- | --- | --- | --- | --- |
|  |  |  |  |  |  |  |  |  |  |  |
|  | **Incubation time (d)** | | | | | | | | |  |
|  | **1** | **2** | **3** | **5** | **7** | **14** | **24** | **34** | **44** | **Strain** |
|  | **log_2_(t_T_/t_0_)** | | | | | | | | |  |
| 1 | -2.82 | -4.31 | -5.63 | -4.34 | -4.44 | -2.74 | -3.66 | -1.96 | -2.78 | H4H18A |
| 2 | -2.73 | -3.32 | -4 | -3.53 | -3.89 | -2.4 | -3.57 | -2.01 | -2.82 | H4L22A |
| 3 | -3.06 | -4.64 | -5.63 | -4.83 | -4.48 | -2.9 | -3.6 | -2.26 | -2.91 | H4H18Q |
| 4 | -2.97 | -4.25 | -5.12 | -3.46 | -4.71 | -2.9 | -3.9 | -2.1 | -2.93 | H4K16Q |
| 5 | -2.72 | -3.99 | -4.82 | -3.65 | -5.05 | -2.83 | -3.94 | -2.23 | -2.98 | H3T80D |
| 6 | -2.66 | -4.9 | -5.48 | -4.42 | -4.77 | -2.5 | -4.85 | -2.68 | -3 | H3E50A |
| 7 | -2.4 | -3.63 | -4.54 | -2.48 | -4.27 | -2.52 | -3.74 | -2.29 | -3.13 | H4K16R |
| 8 | -2.84 | -3.71 | -4.28 | -2.54 | -4.4 | -2.81 | -3.84 | -2.41 | -3.32 | H4[del 9-16] |
| 9 | -4.15 | -5.4 | -6.12 | -5.53 | -5.59 | -3.71 | -4.35 | -2.9 | -3.47 | H4K16A |
| 10 | -3.36 | -4.07 | -5.16 | -4.97 | -4.49 | -2.9 | -3.67 | -2.72 | -3.5 | H4N25D |
| 11 | -3.2 | -3.77 | -4.77 | -4.95 | -4.6 | -2.95 | -4.15 | -2.74 | -3.54 | H3E73A |
| 12 | -2.78 | -3.29 | -4.18 | -3.15 | -3.76 | -2.44 | -4.39 | -3.74 | -3.6 | H3S10A |
| 13 | -2.85 | -3.27 | -4.13 | -4.24 | -4.44 | -2.88 | -4.34 | -2.83 | -3.61 | H3E73Q |
| 14 | -2.58 | -3.21 | -4.08 | -3.58 | -4.15 | -2.47 | -3.97 | -2.81 | -3.61 | H4K59R |
| 15 | -2.63 | -3.73 | -4.94 | -3.48 | -4.67 | -2.72 | -3.98 | -2.89 | -3.62 | H3T80A |
| 16 | -2.14 | -3.11 | -4.18 | -3.32 | -3.81 | -2.28 | -3.91 | -2.78 | -3.62 | H4R19A |
| 17 | -2.48 | -3.17 | -4.2 | -3.6 | -4.33 | -2.76 | -4.35 | -2.97 | -3.76 | H4G94A |
| 18 | -3.72 | -4.59 | -5.38 | -5.82 | -4.84 | -3.27 | -4.11 | -3.18 | -3.94 | H4[del 9-12] |
| 19 | -2.58 | -3.71 | -4.77 | -4.43 | -4.5 | -2.63 | -4.32 | -3.03 | -3.95 | H3P66A |
| 20 | -3.68 | -4.29 | -4.96 | -4.15 | -4.24 | -3.07 | -4.13 | -3.22 | -4.11 | H4R36K |
| 21 | -3.84 | -4.96 | -6.08 | -5.82 | -5.54 | -3.19 | -4.52 | -3.35 | -4.14 | H3E105Q |
| 22 | -3.01 | -4.03 | -5.08 | -3.43 | -4.43 | -2.97 | -4.38 | -3.49 | -4.16 | H3K27A |
| 23 | -3.03 | -4.41 | -5.32 | -4.01 | -4.84 | -3.01 | -4.37 | -3.3 | -4.2 | H3E50Q |
| 24 | -2.72 | -3.26 | -4.18 | -2.84 | -4.33 | -2.96 | -4.47 | -3.44 | -4.2 | H4L10A |
| 25 | -3.57 | -5.04 | -5.94 | -5.13 | -5.24 | -3.43 | -4.34 | -3.38 | -4.22 | H4I50A |
| 26 | -3.22 | -4.97 | -6.05 | -4.04 | -5.23 | -3.58 | -5.21 | -3.72 | -4.22 | H3[del 17-24] |
| 27 | -3.03 | -3.69 | -4.85 | -4.27 | -5.06 | -3.29 | -4.63 | -3.39 | -4.22 | H3Q55E |
| 28 | -2.93 | -4.11 | -5.12 | -3.61 | -4.28 | -2.76 | -4.6 | -3.36 | -4.22 | H3K9R |
| 29 | -2.48 | -3.72 | -4.64 | -3.18 | -4.13 | -2.58 | -4.66 | -3.48 | -4.23 | H3[del 29-32] |
| 30 | -3.07 | -3.58 | -4.5 | -3.52 | -4.26 | -3.04 | -4.32 | -3.53 | -4.25 | H4[del 13-16] |
| 31 | -2.16 | -2.89 | -3.81 | -1.8 | -3.77 | -2.53 | -4.78 | -3.45 | -4.27 | H4G4A |
| 32 | -2.28 | -3.26 | -3.96 | -2.21 | -4.03 | -2.63 | -4.48 | -3.53 | -4.27 | H4G7A |
| 33 | -2.77 | -3.66 | -4.49 | -2.91 | -3.96 | -2.67 | -4.07 | -3.31 | -4.27 | H4[del 5-8] |
| 34 | -2.3 | -3.04 | -4.02 | -2.26 | -3.7 | -2.67 | -4.28 | -3.32 | -4.27 | H4K5A |
| 35 | -4.49 | -5.59 | -5.74 | -4.99 | -4.89 | -3.86 | -4.6 | -3.34 | -4.28 | H4[del 1-12] |
| 36 | -4.45 | -5.16 | -5.58 | -4.62 | -4.46 | -3.7 | -4.65 | -3.46 | -4.28 | H4[del 5-12] |
| 37 | -2.28 | -3.26 | -4.16 | -2.69 | -4.08 | -2.79 | -4.23 | -3.41 | -4.29 | H4S83A |
| 38 | -2.34 | -3.36 | -4.34 | -3.34 | -4.41 | -2.61 | -4.99 | -3.68 | -4.29 | H3R40K |
| 39 | -2.49 | -3.55 | -4.1 | -2.31 | -4.14 | -2.89 | -4.72 | -3.41 | -4.29 | H4G9A |
| 40 | -3.06 | -4.32 | -5.18 | -3.55 | -4.27 | -2.95 | -4.04 | -3.57 | -4.3 | H4F100A |
| 41 | -2.65 | -3.39 | -4.29 | -2.98 | -4.41 | -2.9 | -4.55 | -3.47 | -4.3 | H3R131K |
| 42 | -2.74 | -3.6 | -4.7 | -4.08 | -4.19 | -2.8 | -4.2 | -3.63 | -4.32 | H4H75Q |
| 43 | -2.43 | -2.99 | -4.06 | -3.4 | -4.14 | -2.73 | -4.07 | -3.55 | -4.32 | H3R128K |
| 44 | -2.54 | -3.26 | -4.26 | -3.21 | -4.22 | -2.94 | -4.63 | -3.36 | -4.33 | H3R83K |
| 45 | -2.8 | -3.94 | -4.92 | -3.67 | -4.83 | -2.86 | -4.92 | -3.78 | -4.35 | H3[del 25-28] |
| 46 | -2.64 | -3.42 | -4.69 | -4.44 | -3.84 | -2.87 | -3.99 | -3.62 | -4.35 | H3Q76E |
| 47 | -2.63 | -3.26 | -4.09 | -2.54 | -3.66 | -2.71 | -4.68 | -3.5 | -4.41 | H4K12A |
| 48 | -2.85 | -3.48 | -4.67 | -3.78 | -4.25 | -2.97 | -4.69 | -3.84 | -4.42 | H3K23Q |
| 49 | -2.74 | -3.53 | -4.56 | -3.05 | -4.6 | -3.09 | -4.89 | -3.9 | -4.42 | H3T11D |
| 50 | -3.13 | -4.03 | -5.13 | -4.41 | -4.95 | -3.24 | -4.63 | -3.44 | -4.44 | H3K115R |
| 51 | -2.89 | -3.56 | -4.46 | -2.91 | -4.43 | -3.33 | -4.8 | -3.72 | -4.47 | H3I112A |
| 52 | -2.85 | -3.79 | -4.73 | -3.75 | -4.44 | -3.03 | -4.72 | -3.77 | -4.47 | H3E133A |
| 53 | -2.48 | -3.62 | -4.97 | -3.37 | -4.37 | -2.76 | -4.74 | -3.87 | -4.5 | H4R40K |
| 54 | -3.68 | -4.57 | -5.36 | -4.52 | -5.26 | -3.97 | -4.49 | -3.94 | -4.5 | H3Q55A |
| 55 | -2.3 | -3.35 | -4.3 | -2.38 | -4.16 | -2.67 | -5 | -3.83 | -4.51 | H4K8Q |
| 56 | -2.91 | -3.31 | -4.22 | -3.14 | -4.25 | -3.05 | -4.52 | -3.85 | -4.52 | H4T71A |
| 57 | -3.13 | -4.36 | -5.39 | -4.17 | -4.57 | -3.35 | -4.85 | -4.07 | -4.52 | H3[del 21-28] |
| 58 | -2.47 | -3.14 | -4.17 | -2.5 | -3.89 | -2.88 | -4.52 | -3.78 | -4.53 | H4K8A |
| 59 | -2.2 | -2.64 | -3.51 | -2.31 | -3.65 | -2.74 | -4.81 | -3.85 | -4.53 | H3S22D |
| 60 | -2.65 | -3.58 | -4.9 | -3.23 | -4.47 | -2.9 | -4.95 | -3.78 | -4.53 | H3S28D |
| 61 | -3.27 | -4.15 | -5.36 | -4.63 | -5.26 | -3.43 | -5 | -3.81 | -4.55 | H4P32A |
| 62 | -2.89 | -3.87 | -4.89 | -3.75 | -4.07 | -2.92 | -4.8 | -4.04 | -4.55 | H3[del 25-32] |
| 63 | -2.46 | -3.07 | -3.8 | -3.05 | -3.98 | -2.72 | -4.5 | -3.73 | -4.55 | H4Q27A |
| 64 | -2.39 | -2.82 | -3.68 | -2.28 | -3.84 | -2.79 | -4.98 | -3.77 | -4.56 | H3K27Q |
| 65 | -2.82 | -3.74 | -4.57 | -3.12 | -4.41 | -3.39 | -5.57 | -3.98 | -4.59 | H3T45D |
| 66 | -2.87 | -3.83 | -4.59 | -2.75 | -4.5 | -3.45 | -4.86 | -4.01 | -4.59 | H3T45A |
| 67 | -2.77 | -3.25 | -3.9 | -2.81 | -3.57 | -2.98 | -4.68 | -3.81 | -4.59 | H3T6D |
| 68 | -2.47 | -2.93 | -3.79 | -2 | -3.81 | -2.86 | -5.02 | -3.73 | -4.6 | H4I21A |
| 69 | -3.43 | -4.75 | -5.64 | -3.34 | -5.12 | -3.59 | -5.43 | -4.13 | -4.6 | H3K14Q |
| 70 | -4.22 | -5.47 | -6.37 | -5.48 | -5.73 | -3.85 | -4.71 | -3.79 | -4.61 | H4Y51F |
| 71 | -3.48 | -4.67 | -5.49 | -3.91 | -4.89 | -3.19 | -5.07 | -3.96 | -4.63 | H3K27R |
| 72 | -3.31 | -4.26 | -5.11 | -3.7 | -5.19 | -3.53 | -5.14 | -3.94 | -4.64 | H3L20A |
| 73 | -2.81 | -3.54 | -4.37 | -2.45 | -4.36 | -3.04 | -4.94 | -4 | -4.65 | H4G13A |
| 74 | -2.97 | -3.58 | -4.39 | -3.9 | -4.56 | -3.28 | -4.81 | -3.92 | -4.65 | H4R45K |
| 75 | -2.38 | -3.22 | -4.26 | -2.78 | -4.2 | -2.85 | -5.17 | -3.94 | -4.66 | H3R26K |
| 76 | -3.5 | -4.28 | -5.49 | -5.79 | -5.08 | -3.52 | -4.95 | -3.86 | -4.66 | H3N108A |
| 77 | -2.43 | -3.07 | -4.31 | -3.12 | -3.93 | -2.67 | -5.47 | -4.06 | -4.67 | H3P38V |
| 78 | -2.83 | -4.95 | -6.46 | -5.58 | -6.11 | -3.39 | -4.57 | -4.17 | -4.68 | H3H39Q |
| 79 | -2.83 | -3.47 | -4.22 | -2.45 | -4.26 | -3.21 | -4.83 | -3.79 | -4.69 | H4K5R |
| 80 | -3.47 | -4.65 | -5.64 | -5.13 | -5.42 | -3.58 | -4.73 | -3.97 | -4.69 | H3V96A |
| 81 | -3.06 | -4.02 | -5.04 | -3.43 | -4.59 | -3.28 | -4.61 | -3.57 | -4.69 | H3E59Q |
| 82 | -3.67 | -3.87 | -4.3 | -3.35 | -4.58 | -3.58 | -5.37 | -3.93 | -4.69 | H3F84A |
| 83 | -2.7 | -3.43 | -4.6 | -3.71 | -4.44 | -3.06 | -4.87 | -4.07 | -4.7 | H3Q19E |
| 84 | -2.7 | -3.68 | -4.65 | -3.64 | -4.36 | -3.03 | -5.24 | -3.97 | -4.71 | H3K9A |
| 85 | -3.07 | -3.88 | -4.96 | -4.82 | -5.12 | -3.13 | -5.07 | -4.07 | -4.71 | H4A33S |
| 86 | -2.79 | -3.65 | -4.61 | -4.26 | -4.75 | -2.98 | -4.76 | -4.07 | -4.72 | H3K125Q |
| 87 | -2.8 | -3.73 | -4.76 | -2.66 | -4.34 | -3.18 | -5.08 | -4.25 | -4.74 | H4H75A |
| 88 | -2.93 | -3.49 | -4.57 | -2.91 | -4.2 | -3.12 | -4.9 | -4.03 | -4.75 | H4K12Q |
| 89 | -3.31 | -3.87 | -4.64 | -3.31 | -5.01 | -3.78 | -4.94 | -4.29 | -4.75 | H3R52A |
| 90 | -2.29 | -3.02 | -4.18 | -3.04 | -4.44 | -2.89 | -5.12 | -4.01 | -4.77 | H3S10D |
| 91 | -3.2 | -4.28 | -5.4 | -3.92 | -4.6 | -3.13 | -5 | -3.96 | -4.79 | H3K18R |
| 92 | -2.57 | -3.37 | -4.36 | -3.89 | -3.91 | -2.79 | -4.92 | -4.19 | -4.79 | H4R23A |
| 93 | -4.45 | -4.58 | -4.92 | -1.38 | -3.55 | -4.19 | -5.99 | -4.38 | -4.79 | H4R17A |
| 94 | -3.51 | -4.54 | -5.44 | -4.15 | -4.96 | -3.63 | -4.96 | -3.91 | -4.8 | H3E59A |
| 95 | -2.79 | -3.69 | -4.48 | -2.51 | -4.39 | -3.26 | -4.87 | -4.06 | -4.82 | H4K8R |
| 96 | -2.62 | -3.41 | -4.3 | -2.46 | -4.15 | -2.98 | -5.19 | -4.04 | -4.83 | H4K5Q |
| 97 | -2.82 | -3.59 | -4.66 | -3.73 | -4.38 | -3.35 | -4.92 | -4.07 | -4.84 | H4G14A |
| 98 | -2.9 | -3.64 | -4.95 | -3.7 | -4.66 | -3.16 | -5.37 | -4.26 | -4.84 | H3A21S |
| 99 | -2.85 | -3.31 | -4.39 | -4.25 | -4.62 | -3.05 | -4.88 | -4.09 | -4.85 | H4V65A |
| 100 | -1.98 | -2.77 | -3.29 | -1.64 | -3.39 | -2.41 | -5.41 | -4.14 | -4.86 | H4G6A |
| 101 | -2.36 | -3.07 | -3.75 | -1.51 | -3.58 | -2.88 | -6.14 | -4.1 | -4.86 | H3K79Q |
| 102 | -2.85 | -3.6 | -4.53 | -3.4 | -4.4 | -3.02 | -5.1 | -4.01 | -4.86 | H3G13A |
| 103 | -4.96 | -4.56 | -3.67 | -0.56 | -2.11 | -2.2 | -4.67 | -3.76 | -4.86 | H4L84A |
| 104 | -2.57 | -3.38 | -4.53 | -3.23 | -4.3 | -3.22 | -4.78 | -4.1 | -4.87 | H4T30A |
| 105 | -3.18 | -3.38 | -4.22 | -2.74 | -4.99 | -3.34 | -4.53 | -5.45 | -4.87 | H4D68N |
| 106 | -2.96 | -3.54 | -4.45 | -4 | -4.54 | -3.16 | -4.62 | -4.23 | -4.87 | H4A15S |
| 107 | -2.82 | -3.56 | -4.32 | -2.41 | -4.35 | -3.48 | -5.57 | -4.45 | -4.89 | H3Y41A |
| 108 | -2.54 | -3.39 | -4.63 | -3.66 | -4.49 | -2.93 | -5.36 | -3.99 | -4.9 | H3K9Q |
| 109 | -3.29 | -3.49 | -4.19 | -3.44 | -4.7 | -3.29 | -6.28 | -4.2 | -4.91 | H3D123A |
| 110 | -3.24 | -3.56 | -4.27 | -3.45 | -4.66 | -3.68 | -5.36 | -4.33 | -4.92 | H3Q93A |
| 111 | -2.82 | -3.56 | -4.38 | -3.44 | -4.25 | -2.99 | -4.96 | -4.02 | -4.92 | H3A7S |
| 112 | -2.76 | -3.59 | -4.57 | -3.26 | -4.64 | -3.38 | -5.27 | -4.35 | -4.92 | H3S102D |
| 113 | -3 | -3.11 | -4.08 | -3.71 | -4.23 | -3.13 | -8.18 | -3.94 | -4.92 | H4R45A |
| 114 | -4.03 | -4.73 | -5.32 | -4.89 | -5.58 | -4.2 | -5.34 | -4.19 | -4.93 | H3H113Q |
| 115 | -3.06 | -3.69 | -4.94 | -3.83 | -4.6 | -3.28 | -5.22 | -4.26 | -4.93 | H4E53Q |
| 116 | -2.67 | -3.59 | -4.44 | -2.74 | -4.36 | -3.07 | -4.94 | -4.28 | -4.93 | H4G101A |
| 117 | -2.98 | -3.82 | -4.7 | -3.39 | -4.46 | -3.22 | -5.48 | -4.35 | -4.94 | H3[del 28-31] |
| 118 | -3.21 | -4.27 | -5.25 | -4.15 | -4.67 | -3.45 | -4.99 | -4.25 | -4.94 | H3K23A |
| 119 | -3.11 | -3.59 | -4.74 | -4.1 | -5.16 | -3.58 | -4.75 | -4.55 | -4.95 | H3A15S |
| 120 | -3.42 | -3.77 | -4.32 | -3.29 | -4.64 | -3.72 | -5.57 | -4.26 | -4.97 | H3F67A |
| 121 | -2.77 | -3.61 | -4.75 | -3.65 | -4.51 | -3.09 | -5.09 | -4.21 | -4.98 | H4S47A |
| 122 | -2.79 | -3.38 | -4.37 | -3.15 | -4.2 | -3.22 | -5.65 | -4.45 | -4.99 | H3Y41F |
| 123 | -2.81 | -3.62 | -4.56 | -3.42 | -4.52 | -3.21 | -5.25 | -4.4 | -5 | H3Y99F |
| 124 | -2.45 | -3.21 | -4.21 | -2.54 | -4.03 | -3.09 | -4.92 | -4.07 | -5 | H4G2A |
| 125 | -2.52 | -3.29 | -4.29 | -4.52 | -4.56 | -3.27 | -5.06 | -4.19 | -5.01 | H4K31Q |
| 126 | -3.12 | -4.06 | -5.1 | -3.76 | -4.74 | -3.22 | -5.55 | -4.28 | -5.01 | H3K23R |
| 127 | -2.91 | -3.67 | -4.82 | -4.21 | -4.84 | -3.19 | -5.11 | -4.06 | -5.02 | H3S57A |
| 128 | -3.12 | -4.03 | -5.02 | -4.27 | -4.62 | -3.52 | -4.8 | -4.21 | -5.03 | H4V70A |
| 129 | -3.13 | -4.01 | -5.37 | -3.9 | -5.19 | -3.57 | -5.19 | -4.56 | -5.03 | H3R17A |
| 130 | -3.59 | -4.06 | -4.46 | -3.63 | -4.8 | -3.91 | -5.38 | -4.37 | -5.04 | H4Y51E |
| 131 | -2.58 | -3.21 | -4.63 | -4.18 | -4.63 | -2.8 | -5.26 | -4.3 | -5.04 | H3D81N |
| 132 | -2.88 | -3.63 | -4.58 | -3.64 | -4.49 | -3.35 | -5.25 | -4.32 | -5.04 | pJD47_H3_wild-type |
| 133 | -2.49 | -3.07 | -4.21 | -3.83 | -4.43 | -2.74 | -5.05 | -4.03 | -5.04 | H3R128A |
| 134 | -3.56 | -3.71 | -4.35 | -3.18 | -4.73 | -3.7 | -5.65 | -4.21 | -5.05 | H3I62A |
| 135 | -3.03 | -3.89 | -5.03 | -3.84 | -4.88 | -3.61 | -5.28 | -4.41 | -5.05 | H3R26A |
| 136 | -2.09 | -2.46 | -3.52 | -2.62 | -4.05 | -2.76 | -6.59 | -4.36 | -5.06 | H4G102A |
| 137 | -3.77 | -4.17 | -4.83 | -3.67 | -4.88 | -3.92 | -5.28 | -4.39 | -5.07 | H4T73D |
| 138 | -2.6 | -3.11 | -4.11 | -3.95 | -4.15 | -3 | -5.29 | -4.39 | -5.07 | H4I26A |
| 139 | -2.88 | -3.61 | -4.94 | -4.59 | -4.93 | -3.33 | -5.21 | -4.27 | -5.07 | H3K121R |
| 140 | -2.77 | -3.4 | -4.67 | -3.94 | -4.38 | -3.17 | -5.14 | -4.38 | -5.07 | H3S135D |
| 141 | -3.32 | -3.72 | -4.47 | -3.55 | -4.87 | -3.82 | -5.63 | -4.38 | -5.08 | H3I124A |
| 142 | -2.98 | -3.7 | -4.34 | -2.71 | -4.38 | -3.43 | -5.27 | -4.25 | -5.08 | H4G11A |
| 143 | -3.58 | -3.82 | -4.55 | -3.62 | -4.89 | -3.84 | -5.47 | -4.4 | -5.1 | H4T80D |
| 144 | -4.14 | -4.85 | -5.52 | -4.36 | -5.72 | -4.28 | -5.46 | -4.5 | -5.11 | H3[del 1-36] |
| 145 | -3.75 | -4.23 | -4.62 | -3.96 | -4.95 | -3.85 | -5.45 | -4.42 | -5.11 | H3T107D |
| 146 | -3.3 | -3.96 | -4.61 | -3.44 | -4.89 | -3.79 | -5.17 | -4.48 | -5.11 | H3N108D |
| 147 | -2.48 | -3.22 | -4.25 | -2.39 | -4.16 | -3.02 | -5.2 | -4.46 | -5.11 | H4Y98F |
| 148 | -3.92 | -4.1 | -3.94 | -1.92 | -3.9 | -3.68 | -5.64 | -4.28 | -5.11 | H3E97A |
| 149 | -3.61 | -3.95 | -4.32 | -3.36 | -4.71 | -3.77 | -5.43 | -4.55 | -5.11 | H4I34A |
| 150 | -2.7 | -3.17 | -4.1 | -2.62 | -4.17 | -3.32 | -5.03 | -4.42 | -5.11 | H3A25S |
| 151 | -3.87 | -4.16 | -4.68 | -4.12 | -5.03 | -3.85 | -5.37 | -4.28 | -5.12 | H3R116K |
| 152 | -2.79 | -3.32 | -4.33 | -3.17 | -4.39 | -3.2 | -5.59 | -4.3 | -5.12 | H4K20R |
| 153 | -2.57 | -3.07 | -4.17 | -3.48 | -4.29 | -3.01 | -5.22 | -4.53 | -5.13 | H3T6A |
| 154 | -2.66 | -3.06 | -4.09 | -3.8 | -4.55 | -3.23 | -5.34 | -4.27 | -5.13 | H3G132A |
| 155 | -2.13 | -2.93 | -4.03 | -2.94 | -3.65 | -2.74 | -5.17 | -4.48 | -5.14 | H4R3A |
| 156 | -2.63 | -3.48 | -4.61 | -4.38 | -4.7 | -3.39 | -5.7 | -4.55 | -5.18 | H3K125A |
| 157 | -3.35 | -3.65 | -4.24 | -2.84 | -4.6 | -3.82 | -6.17 | -4.39 | -5.18 | H3[del 4-10] |
| 158 | -3.67 | -4.31 | -5.2 | -5.93 | -5.38 | -3.81 | -4.84 | -4.49 | -5.19 | H4K31A |
| 159 | -2.11 | -2.77 | -4.2 | -4.22 | -4.21 | -2.94 | -6.16 | -4.49 | -5.19 | H3V101A |
| 160 | -3.66 | -4.92 | -5.88 | -4.1 | -5.08 | -3.91 | -5.59 | -4.41 | -5.19 | H3[del 21-32] |
| 161 | -3.08 | -3.54 | -4.71 | -4.63 | -4.49 | -3.46 | -5.45 | -4.42 | -5.19 | H4N25A |
| 162 | -3.73 | -3.87 | -4.51 | -3.26 | -5.09 | -3.99 | -5.63 | -4.37 | -5.21 | H3L103A |
| 163 | -3.26 | -4.08 | -5.06 | -3.73 | -4.82 | -3.68 | -5.34 | -4.57 | -5.22 | H3S22A |
| 164 | -3.76 | -3.92 | -4.76 | -2.89 | -5.05 | -3.7 | -5.26 | -4.39 | -5.23 | H3E94Q |
| 165 | -2.56 | -3.01 | -3.96 | -3.02 | -4.28 | -3.13 | -5.07 | -4.55 | -5.24 | H3S31D |
| 166 | -2.89 | -3.41 | -4.33 | -3.92 | -4.31 | -3.26 | -5.14 | -4.56 | -5.24 | H4K20Q |
| 167 | -4.31 | -5.22 | -5.54 | -3.66 | -4.39 | -3.9 | -5.31 | -4.46 | -5.24 | H3D106A |
| 168 | -3.77 | -4.48 | -5.27 | -4.49 | -5.05 | -3.95 | -5.55 | -4.61 | -5.25 | H3L48A |
| 169 | -3.31 | -3.95 | -5.04 | -4.33 | -5.01 | -3.59 | -5.29 | -4.45 | -5.26 | H4S69A |
| 170 | -3.84 | -5.1 | -5.93 | -3.31 | -5 | -3.75 | -5.72 | -4.55 | -5.27 | H3K14R |
| 171 | -3.61 | -4.21 | -4.79 | -4.27 | -5.14 | -4 | -5.29 | -4.66 | -5.27 | H3Q120E |
| 172 | -2.84 | -3.53 | -4.59 | -3.35 | -4.59 | -3.27 | -5.12 | -4.38 | -5.27 | H3K79R |
| 173 | -3.86 | -4.3 | -4.59 | -3.65 | -5 | -3.98 | -5.81 | -4.63 | -5.27 | H4A76S |
| 174 | -2.89 | -3.25 | -4.3 | -3.73 | -4.34 | -3.22 | -4.95 | -4.52 | -5.27 | H3D77N |
| 175 | -3.15 | -3.91 | -4.91 | -4.18 | -4.73 | -3.44 | -5.4 | -4.49 | -5.29 | H3T11A |
| 176 | -3.1 | -5.26 | -6.36 | -5.76 | -5.13 | -4.15 | -6.12 | -4.65 | -5.31 | H4[del 13-20] |
| 177 | -2.76 | -3.58 | -4.68 | -4.05 | -4.6 | -3.33 | -5.34 | -4.39 | -5.31 | H3P43A |
| 178 | -3.58 | -4 | -4.56 | -3.83 | -4.69 | -3.73 | -5.82 | -4.47 | -5.31 | H3I119A |
| 179 | -2.65 | -3.33 | -4.24 | -3.01 | -4.23 | -3.22 | -5.49 | -4.55 | -5.31 | H3S102A |
| 180 | -3.51 | -3.97 | -4.69 | -3.47 | -4.91 | -4 | -5.32 | -4.58 | -5.32 | H4L90A |
| 181 | -3.72 | -5.44 | -5.85 | -5.18 | -4.67 | -4.65 | -5.77 | -4.98 | -5.33 | H4[del 9-20] |
| 182 | -3.18 | -4.06 | -5.26 | -4.25 | -5.18 | -3.54 | -5.52 | -4.4 | -5.33 | H3R129A |
| 183 | -3.13 | -5.25 | -6.29 | -4.8 | -5.72 | -3.43 | -5.11 | -5.19 | -5.34 | H3[del 25-36] |
| 184 | -3.04 | -3.99 | -4.92 | -4.25 | -4.88 | -3.51 | -6.12 | -4.8 | -5.35 | H3D81A |
| 185 | -2.94 | -3.69 | -4.89 | -4.27 | -4.68 | -3.64 | -4.68 | -4.83 | -5.35 | H4S1D |
| 186 | -3.82 | -5.32 | -6.18 | -4.48 | -5.3 | -3.91 | -5.49 | -4.78 | -5.35 | H4R39K |
| 187 | -3.27 | -4.21 | -5.25 | -3.56 | -5.27 | -3.81 | -6.3 | -4.89 | -5.36 | H3R17K |
| 188 | -4.57 | -5.12 | -5.77 | -4.83 | -3.99 | -4.18 | -5.65 | -4.98 | -5.38 | H4[del 20-23] |
| 189 | -4.09 | -4.39 | -4.82 | -4.36 | -5.23 | -4.15 | -6 | -4.64 | -5.39 | H3D123N |
| 190 | -2.5 | -3.18 | -4.32 | -3.25 | -4.31 | -3.25 | -5.64 | -4.86 | -5.4 | H3S95A |
| 191 | -3.15 | -3.79 | -4.69 | -3.95 | -4.85 | -3.55 | -5.55 | -4.59 | -5.4 | H3K37R |
| 192 | -2.81 | -3.97 | -4.97 | -4.29 | -4.75 | -3.74 | -5.48 | -4.68 | -5.4 | H4A38S |
| 193 | -3.43 | -4.62 | -5.89 | -3.93 | -5.27 | -3.82 | -5.67 | -4.77 | -5.4 | H3K14A |
| 194 | -2.7 | -3.13 | -4.1 | -3.23 | -4.13 | -3.32 | -5.95 | -4.7 | -5.41 | H4R19K |
| 195 | -2.62 | -3.31 | -4.25 | -2.99 | -4.32 | -3.29 | -6.32 | -4.94 | -5.41 | H3V35A |
| 196 | -3.23 | -4.09 | -5.16 | -4.01 | -4.88 | -3.69 | -5.6 | -4.75 | -5.41 | H3A29S |
| 197 | -2.48 | -3.27 | -4.06 | -2.22 | -4.47 | -3.3 | -6 | -4.64 | -5.41 | H4T96D |
| 198 | -2.92 | -3.61 | -4.65 | -4.21 | -4.85 | -3.46 | -5.52 | -4.71 | -5.41 | H3R131A |
| 199 | -3.16 | -4.4 | -5.56 | -4.66 | -5.08 | -3.67 | -5.34 | -4.94 | -5.42 | H3K18A |
| 200 | -2.9 | -3.51 | -4.48 | -3.63 | -4.62 | -3.4 | -5.52 | -4.65 | -5.42 | H4S60D |
| 201 | -3.76 | -4.07 | -4.81 | -4 | -4.82 | -3.91 | -5.3 | -4.54 | -5.42 | H4R39A |
| 202 | -2.84 | -3.59 | -4.47 | -4.29 | -4.25 | -3.11 | -5.17 | -4.6 | -5.42 | H3R134A |
| 203 | -2.89 | -3.68 | -4.63 | -3.42 | -4.83 | -3.27 | -5.49 | -4.52 | -5.43 | H3G12A |
| 204 | -2.78 | -3.3 | -4.35 | -3.86 | -4.47 | -3.4 | -5.81 | -5.11 | -5.43 | H3T32A |
| 205 | -2.83 | -3.44 | -4.43 | -3.43 | -4.44 | -3.32 | -5.72 | -4.65 | -5.43 | H3[del 21-24] |
| 206 | -2.76 | -3.29 | -4.43 | -3.34 | -4.71 | -3.39 | -6.23 | -4.65 | -5.44 | H3A98S |
| 207 | -3.79 | -4.58 | -5.85 | -5.04 | -5.02 | -4.02 | -5.24 | -4.4 | -5.44 | H4E52Q |
| 208 | -2.81 | -3.57 | -4.61 | -4.55 | -4.64 | -3.54 | -5.66 | -4.63 | -5.44 | H3K64R |
| 209 | -2.9 | -3.73 | -5.46 | -3.93 | -4.86 | -3.38 | -5.59 | -4.65 | -5.45 | H3K79A |
| 210 | -3.01 | -3.79 | -4.87 | -4.11 | -4.58 | -3.54 | -5.57 | -4.76 | -5.46 | H4A56S |
| 211 | -3.93 | -4.1 | -5.09 | -4.32 | -5.61 | -4.02 | -5.81 | -4.82 | -5.46 | H3R116A |
| 212 | -2.91 | -3.29 | -4.2 | -3.64 | -4.52 | -3.38 | -5.7 | -4.79 | -5.46 | H4K79Q |
| 213 | -3.45 | -4.19 | -5.19 | -5.23 | -5.26 | -3.91 | -5.72 | -4.77 | -5.46 | H4V57A |
| 214 | -5.98 | -5.37 | -4.57 | -4.71 | -2.67 | -3.41 | -5.92 | -4.61 | -5.47 | H3G90A |
| 215 | -3.59 | -3.8 | -4.57 | -3.52 | -4.94 | -4.15 | -5.65 | -4.92 | -5.48 | H4Y72F |
| 216 | -3.66 | -3.86 | -4.45 | -3.05 | -4.79 | -4.26 | -5.7 | -4.72 | -5.49 | H4D85N |
| 217 | -3.94 | -4.99 | -5.82 | -5.57 | -5.71 | -4.07 | -5.65 | -4.83 | -5.5 | H3E133Q |
| 218 | -2.44 | -2.88 | -3.9 | -3.3 | -4.18 | -2.9 | -5.37 | -4.82 | -5.51 | H3R72K |
| 219 | -3.46 | -4.4 | -5.14 | -3.87 | -4.96 | -4.28 | -5.73 | -4.99 | -5.53 | H3Y41E |
| 220 | -3.97 | -4.67 | -5.49 | -3.89 | -5.43 | -4.67 | -5.1 | -4.92 | -5.53 | H3E97Q |
| 221 | -2.45 | -3.09 | -4.14 | -3.44 | -3.82 | -3.36 | -5.4 | -4.91 | -5.53 | H4R23K |
| 222 | -2.57 | -3.33 | -4.6 | -3.77 | -4.26 | -3.07 | -5.76 | -4.94 | -5.53 | H3A91S |
| 223 | -4.8 | -4.43 | -4.98 | -3.94 | -5.35 | -4.4 | -6.68 | -4.94 | -5.53 | H4[del 21-24] |
| 224 | -2.99 | -3.68 | -4.76 | -4.24 | -4.64 | -3.55 | -5.43 | -4.8 | -5.55 | H3Q19A |
| 225 | -2.84 | -3.49 | -4.53 | -3.79 | -4.6 | -3.41 | -5.73 | -4.92 | -5.56 | H3S135A |
| 226 | -3.81 | -4.21 | -4.87 | -4.1 | -5.21 | -4.22 | -6.32 | -4.62 | -5.58 | H3L130A |
| 227 | -2.38 | -2.99 | -3.95 | -3.22 | -4.15 | -3.06 | -5.82 | -4.72 | -5.58 | H4S64A |
| 228 | -3.24 | -4.69 | -5.59 | -3.51 | -5.47 | -3.89 | -5.98 | -4.81 | -5.58 | H3[del 17-20] |
| 229 | -3.38 | -3.98 | -4.89 | -4.03 | -5.05 | -3.81 | -5.25 | -4.83 | -5.59 | H4G41A |
| 230 | -3.15 | -3.37 | -4.31 | -3.55 | -4.07 | -3.55 | -5.91 | -4.88 | -5.59 | H4K20A |
| 231 | -3.03 | -3.49 | -4.62 | -4.34 | -4.78 | -3.51 | -5.57 | -4.61 | -5.59 | H3A47S |
| 232 | -2.63 | -3.12 | -3.94 | -3.67 | -4.23 | -3.38 | -5.63 | -5.01 | -5.59 | H4Q27E |
| 233 | -2.69 | -2.97 | -3.83 | -4.51 | -3.98 | -3.26 | -5.81 | -4.93 | -5.6 | H3L70A |
| 234 | -2.61 | -3.25 | -4.05 | -2.98 | -4.51 | -3.32 | -5.52 | -4.79 | -5.6 | H4K79R |
| 235 | -2.88 | -3.42 | -4.55 | -4.58 | -4.81 | -3.74 | -5.72 | -4.89 | -5.61 | H4K77R |
| 236 | -4.04 | -4.4 | -4.75 | -3.38 | -5.15 | -4.04 | -5.86 | -4.71 | -5.61 | H4K91Q |
| 237 | -2.71 | -3.57 | -4.61 | -3.88 | -4.52 | -3.35 | -5.82 | -4.97 | -5.62 | H3T32D |
| 238 | -3.57 | -5.13 | -6.42 | -5.02 | -5.12 | -3.84 | -5.77 | -5.75 | -5.65 | H3[del 29-36] |
| 239 | -3.13 | -3.84 | -4.91 | -4.12 | -4.89 | -3.7 | -5.7 | -5.09 | -5.66 | H3P38A |
| 240 | -4.16 | -5.01 | -5.33 | -4.91 | -5.48 | -4.32 | -6.03 | -5.03 | -5.66 | H3T118A |
| 241 | -3.26 | -3.97 | -4.79 | -3.15 | -4.51 | -3.39 | -5.87 | -4.62 | -5.68 | H4T71D |
| 242 | -2.7 | -2.92 | -4.04 | -2.65 | -4.24 | -3.11 | -5.29 | -4.97 | -5.68 | H4T96A |
| 243 | -2.98 | -3.46 | -4.37 | -3.78 | -4.51 | -3.44 | -5.63 | -4.94 | -5.71 | H4S1A |
| 244 | -3.2 | -4.02 | -5.05 | -3.62 | -5.01 | -3.64 | -6.12 | -5.25 | -5.71 | H3P16A |
| 245 | -3.49 | -4.51 | -5.62 | -5.42 | -5.59 | -4.02 | -5.5 | -4.86 | -5.75 | H4V43A |
| 246 | -3.63 | -3.87 | -4.44 | -3.43 | -4.91 | -4 | -5.98 | -4.82 | -5.75 | H3Q68E |
| 247 | -3.8 | -5.05 | -6.22 | -4.21 | -4.72 | -3.72 | -6.06 | -5.44 | -5.75 | H3K36R |
| 248 | -2.71 | -3.32 | -4.15 | -2.83 | -4.73 | -3.2 | -7.49 | -4.85 | -5.75 | H3R8K |
| 249 | -2.81 | -3.5 | -4.66 | -4.01 | -4.71 | -3.45 | -6.02 | -5.17 | -5.77 | H3S31A |
| 250 | -2.99 | -3.9 | -4.99 | -4.86 | -4.77 | -3.38 | -5.6 | -4.93 | -5.78 | H4K91R |
| 251 | -3.98 | -5.38 | -6.99 | -4.94 | -5.36 | -4.11 | -6.46 | -5.88 | -5.78 | H3K4,9,14,18Q |
| 252 | -3.64 | -4.62 | -5.96 | -3.79 | -5.13 | -4.07 | -6.35 | -5.15 | -5.78 | H3[del 9-12] |
| 253 | -4.39 | -4.79 | -5.24 | -4.27 | -5.63 | -4.63 | -6.55 | -5.01 | -5.8 | H3[del 5-36] |
| 254 | -2.99 | -3.68 | -4.72 | -4.08 | -4.62 | -3.53 | -5.77 | -5.08 | -5.81 | H4S60A |
| 255 | -2.53 | -3.17 | -4.03 | -4.29 | -4.23 | -3.49 | -5.97 | -5.19 | -5.82 | H3R72A |
| 256 | -3.42 | -4.25 | -5.45 | -5.73 | -4.51 | -3.65 | -5.97 | -5.02 | -5.83 | H4K59Q |
| 257 | -2.63 | -3.03 | -3.97 | -2.95 | -4.35 | -3.39 | -6.03 | -5.16 | -5.83 | H4Q93A |
| 258 | -3.26 | -4.07 | -5 | -3.27 | -4.91 | -3.97 | -5.94 | -5.02 | -5.84 | H3E94A |
| 259 | -2.91 | -3.6 | -4.6 | -3.84 | -4.54 | -3.53 | -5.93 | -5.31 | -5.87 | H3P30A |
| 260 | -3.53 | -3.93 | -4.6 | -2.4 | -4.93 | -4.21 | -6.04 | -5.22 | -5.89 | H3R52K |
| 261 | -2.72 | -3.09 | -4.26 | -3.3 | -4.21 | -3.28 | -6.26 | -5.16 | -5.89 | H4R92K |
| 262 | -2.8 | -3.28 | -4.04 | -3.11 | -4.11 | -3.32 | -5.7 | -5.14 | -5.89 | H4R55K |
| 263 | -3.94 | -4.82 | -5.91 | -4.66 | -5.73 | -4.54 | -6.29 | -5.32 | -5.89 | H3L109A |
| 264 | -4.17 | -5.93 | -6.5 | -3.89 | -5.52 | -4.05 | -6.22 | -5.32 | -5.9 | H3[del 9-16] |
| 265 | -2.87 | -3.49 | -4.54 | -4.08 | -4.68 | -3.47 | -7.91 | -5.04 | -5.9 | H4R67K |
| 266 | -3.5 | -4.2 | -4.84 | -3.65 | -5.07 | -4.01 | -6.06 | -5.42 | -5.92 | H3R134K |
| 267 | -2.92 | -3.86 | -4.72 | -5.35 | -4.95 | -3.9 | -5.88 | -5.12 | -5.93 | H3Y99A |
| 268 | -2.99 | -3.42 | -4.34 | -4.5 | -4.44 | -3.86 | -6.66 | -5.64 | -5.95 | H3Q76A |
| 269 | -3.64 | -5.18 | -6.24 | -5.49 | -5.12 | -3.82 | -6.32 | -5.05 | -5.96 | H3D106N |
| 270 | -3.29 | -4.14 | -5.67 | -5.34 | -5.15 | -3.98 | -5.66 | -5.37 | -5.98 | H3H39A |
| 271 | -2.99 | -3.49 | -4.4 | -3.59 | -4.5 | -3.57 | -6.3 | -5.5 | -6 | H4E63Q |
| 272 | -4.2 | -4.55 | -5.01 | -3.81 | -5.55 | -4.71 | -6.45 | -5.37 | -6 | H4S69D |
| 273 | -2.65 | -3.21 | -4.12 | -3.32 | -3.92 | -3.28 | -6.12 | -5.47 | -6 | H4R95K |
| 274 | -3.3 | -3.82 | -4.87 | -4.31 | -5.14 | -3.89 | -5.69 | -5.36 | -6 | H3A24S |
| 275 | -2.68 | -3.05 | -4.24 | -4.63 | -4.29 | -3.66 | -6.09 | -5.13 | -6.01 | H3A1S |
| 276 | -3.64 | -4.01 | -4.74 | -3.86 | -5.1 | -4.26 | -6.09 | -5.22 | -6.03 | H4Y72A |
| 277 | -4.98 | -5.61 | -6.57 | -5.88 | -6.47 | -5.18 | -6.3 | -5.44 | -6.04 | H3I51A |
| 278 | -4.23 | -4.58 | -5.22 | -3.69 | -5.79 | -4.64 | -5.74 | -5.53 | -6.05 | H4S83D |
| 279 | -3.19 | -4.2 | -5.44 | -5.51 | -5.08 | -3.83 | -5.81 | -5.25 | -6.09 | H3K37Q |
| 280 | -3.15 | -4.96 | -5.23 | -4.46 | -4.27 | -3.42 | -8.28 | -6.42 | -6.1 | H3[del 32-35] |
| 281 | -2.81 | -3.32 | -4.66 | -4.09 | -4.32 | -3.46 | -5.64 | -5.31 | -6.11 | H3G34A |
| 282 | -2.59 | -3.15 | -4.01 | -2.99 | -4.34 | -3.56 | -6.06 | -5.49 | -6.12 | H4Q93E |
| 283 | -3.56 | -4.42 | -5.5 | -5.01 | -4.69 | -3.9 | -7.06 | -5.91 | -6.13 | H4R17K |
| 284 | -6.58 | -7.25 | -6.81 | -6.25 | -4.82 | -5.17 | -6.13 | -5.59 | -6.14 | H4K5,8,12,16Q |
| 285 | -3.69 | -5.04 | -6.5 | -5.18 | -5.91 | -4.43 | -5.77 | -5.31 | -6.15 | H3R40A |
| 286 | -3.15 | -3.71 | -4.75 | -4.84 | -5.08 | -4.01 | -6.54 | -5.5 | -6.15 | H3K64A |
| 287 | -3.71 | -3.94 | -4.61 | -3.79 | -4.59 | -4.19 | -6.27 | -5.26 | -6.15 | H3D77A |
| 288 | -3.1 | -3.45 | -4.5 | -4.66 | -4.71 | -3.72 | -6.27 | -5.24 | -6.16 | H3K125R |
| 289 | -6.15 | -6.38 | -5.88 | -5 | -3.92 | -4.07 |  | -5.46 | -6.17 | H4K5,8,12,16A |
| 290 | -3.31 | -3.62 | -4.35 | -2.96 | -4.41 | -3.82 | -6.16 | -5.45 | -6.21 | H3A75S |
| 291 | -2.79 | -3.38 | -4.39 | -3.18 | -4.66 | -3.58 | -6.62 | -5.57 | -6.22 | H4R67A |
| 292 | -2.47 | -3.06 | -4.34 | -3.78 | -4.4 | -3.51 | -6.23 | -5.62 | -6.22 | H4R95A |
| 293 | -3.89 | -4.7 | -5.22 | -3.21 | -5.05 | -4.14 | -6.62 | -5.48 | -6.24 | H3[del 13-16] |
| 294 | -2.92 | -3.82 | -4.93 | -4.39 | -4.75 | -3.77 | -6.67 | -5.51 | -6.25 | H3A127S |
| 295 | -3.03 | -3.15 | -4.65 | -4.83 | -4.61 | -3.57 | -6.2 | -5.65 | -6.25 | H4V87A |
| 296 | -3.26 | -4.01 | -5.11 | -4.07 | -4.74 | -3.74 | -6.25 | -5.16 | -6.25 | H3G33A |
| 297 | -2.75 | -3.16 | -4.22 | -4.4 | -4.62 | -3.6 | -6.81 | -5.77 | -6.29 | H3K121A |
| 298 | -2.67 | -3.15 | -4.09 | -3.69 | -4.32 | -3.52 | -6.78 | -5.99 | -6.3 | H3P30V |
| 299 | -2.8 | -3.21 | -4.22 | -3.63 | -4.47 | -3.38 | -6.7 | -5.69 | -6.34 | H4E63A |
| 300 | -2.97 | -3.11 | -3.82 | -3.91 | -4.25 | -3.53 | -6.56 | -5.37 | -6.35 | H4D24A |
| 301 | -2.68 | -3.42 | -4.57 | -4.01 | -4.93 | -3.96 | -6.41 | -5.73 | -6.35 | H3R8A |
| 302 | -3.65 | -4.01 | -4.49 | -3.53 | -4.77 | -4.41 | -6.38 | -5.7 | -6.36 | H4S64D |
| 303 | -3.55 | -4.96 | -6.21 | -5.36 | -4.92 | -3.87 | -6.48 | -6.52 | -6.39 | H3K36Q |
| 304 | -3.5 | -3.95 | -4.98 | -4.32 | -4.96 | -3.89 | -6.52 | -5.56 | -6.39 | H3K42R |
| 305 | -4.99 | -5.65 | -5.3 | -4.42 | -4.97 | -4.03 | -8.8 | -5.41 | -6.4 | H4[del 5-16] |
| 306 | -2.93 | -3.32 | -4.74 | -3.93 | -4.52 | -4.02 | -6.68 | -6.09 | -6.42 | H4K77A |
| 307 | -3.25 | -3.6 | -4.69 | -4.79 | -4.83 | -4.02 | -6.6 | -5.64 | -6.43 | H4D24N |
| 308 | -3.55 | -4.21 | -5.03 | -4.85 | -4.83 | -3.75 | -10.33 | -5.81 | -6.45 | H4D68A |
| 309 | -3.63 | -4.51 | -5.87 | -6.48 | -5.49 | -4.16 | -6.24 | -5.84 | -6.54 | H4Y88F |
| 310 | -5.65 | -5.92 | -5.77 | -5.25 | -5.68 | -5.34 | -7.09 | -5.93 | -6.56 | H4[del 5-20] |
| 311 | -3.84 | -4.53 | -5.26 | -5.18 | -5.58 | -4.81 | -6.47 | -5.65 | -6.57 | H4L62A |
| 312 | -5.17 | -5.89 | -7.02 | -7.1 | -6.81 | -5.38 | -6.16 | -5.42 | -6.6 | H3S57D |
| 313 | -3.19 | -3.55 | -4.59 | -4.17 | -4.91 | -3.93 | -6.27 | -5.61 | -6.66 | H3T58A |
| 314 | -3.91 | -5.49 | -6.45 | -5.75 | -5.52 | -4.93 | -6.96 | -5.79 | -6.67 | H4[del 17-20] |
| 315 | -5.17 | -5.42 | -5.38 | -4.53 | -5.57 | -5.12 | -7.31 | -5.97 | -6.72 | H4[del 4-19] |
| 316 | -3.59 | -4 | -4.61 | -3.29 | -4.43 | -4.64 | -6.76 | -5.97 | -6.75 | H4K12R |
| 317 | -4.16 | -4.89 | -5.86 | -5.45 | -6.31 | -4.53 | -7.18 | -5.77 | -6.75 | H4Y88A |
| 318 | -4.29 | -4.44 | -5.53 | -5.72 | -5.56 | -4.26 | -6.46 | -5.81 | -6.8 | H3L65A |
| 319 | -3.59 | -3.73 | -4.13 | -4.21 | -4.34 | -4.2 | -6.05 | -5.86 | -6.84 | H3S28A |
| 320 | -3.43 | -3.92 | -4.92 | -4.68 | -5.48 | -4.38 | -6.71 | -6.12 | -6.84 | H4E74A |
| 321 | -3.47 | -4.57 | -6.36 | -5.79 | -4.56 | -4.06 | -7.4 | -7.12 | -6.88 | H3[del 33-36] |
| 322 | -5.25 | -6.49 | -6.89 | -6.57 | -6.12 | -6.01 | -8 | -6.76 | -6.95 | H4[del 13-24] |
| 323 | -2.66 | -3.16 | -4.28 | -3.82 | -4.44 | -3.52 | -6.2 | -5.93 | -6.99 | H4R92A |
| 324 | -3.06 | -3 | -3.53 | -3.26 | -3.34 | -4.05 | -6.28 | -6.36 | -7.02 | H4G99A |
| 325 | -3.28 | -3.39 | -4.34 | -4.32 | -4.56 | -4.03 | -6.13 | -6.2 | -7.07 | H3K121Q |
| 326 | -3.69 | -4.27 | -5.21 | -5.09 | -5.56 | -4.41 | -7.14 | -6.69 | -7.07 | H3S87A |
| 327 | -3.37 | -3.99 | -5.17 | -5.8 | -5.38 | -4.79 | -7.58 | -6.77 | -7.12 | H3K64Q |
| 328 | -3.39 | -4.89 | -5.79 | -5.01 | -5.63 | -4.22 | -6.54 | -6.2 | -7.16 | H4K44R |
| 329 | -3.67 | -4.8 | -5.92 | -5.61 | -5.57 | -4.46 | -7.04 | -6.88 | -7.21 | H3K37A |
| 330 | -3.69 | -4.08 | -5.63 | -6.26 | -5.36 | -4.55 | -6.93 | -6.2 | -7.23 | H3A114S |
| 331 | -3.1 | -3.28 | -3.98 | -3.19 | -4.28 | -3.95 | -6.99 | -6.49 | -7.27 | H4K77Q |
| 332 | -6.77 | -6.55 | -7.97 | -6.45 | -8.16 | -6.25 | -8.21 | -6.45 | -7.27 | H3K115Q |
| 333 | -3.03 | -3.91 | -5.19 | -4.79 | -4.76 | -4.39 | -7.22 | -6.63 | -7.3 | H3R49K |
| 334 | -4.6 | -5.12 | -6.08 | -5.2 | -5.4 | -4.63 | -6.78 | -6.56 | -7.34 | H3R69A |
| 335 | -5.25 | -5.73 | -5.64 | -4.86 | -3.37 | -3.86 | -7.58 | -6.84 | -7.36 | H4[del 17-24] |
| 336 | -4.77 | -3.48 | -3.65 | -5.32 | -7.16 | -5.03 | -9.02 | -8.62 | -7.36 | H4S47D |
| 337 | -3.17 | -3.43 | -4.54 | -3.85 | -4.72 | -4.58 | -7.03 | -7.29 | -7.37 | H4T82A |
| 338 | -3.05 | -3.44 | -4.82 | -4.48 | -5.02 | -4.15 | -7.8 | -6.69 | -7.37 | H3A88S |
| 339 | -3.61 | -3.85 | -4.93 | -4.59 | -5.15 | -4.87 | -7.04 | -6.92 | -7.48 | H4L58A |
| 340 | -2.89 | -2.98 | -4.35 | -4.04 | -4.5 | -4.42 | -7.57 | -7.15 | -7.53 | H3Q85E |
| 341 | -3.4 | -3.85 | -5.07 | -5.52 | -5.17 | -4.68 | -7.54 | -6.95 | -7.54 | H3A111S |
| 342 | -6.96 | -7.06 | -10.16 | -6.1 | -8.35 | -7.44 | -8.21 | -8.81 | -7.55 | H3K4,9,14,18A |
| 343 | -5.02 | -6.35 | -7.14 | -6.44 | -7.01 | -5.45 | -7.78 | -6.69 | -7.56 | H4E52A |
| 344 | -6.64 | -8.02 | -8.26 | -7.92 | -7.38 | -6.27 | -7.17 | -6.67 | -7.67 | H4R36A |
| 345 | -3.83 | -4.54 | -6.06 | -6.34 | -5.95 | -5.42 | -7.04 | -6.37 | -7.7 | H4G42A |
| 346 | -6.29 | -6.43 | -6.28 | -6.26 | -6.68 | -5.92 | -8.47 | -6.75 | -7.71 | H4[del 5-24] |
| 347 | -6.34 | -6.67 | -6.71 | -5.94 | -6.69 | -6.29 | -7.48 | -7.17 | -7.75 | H4[del 4-23] |
| 348 | -4.65 | -6.08 | -7.75 | -5 | -6.28 | -5.84 | -8.35 | -7.8 | -7.82 | H3[del 13-20] |
| 349 | -3.63 | -3.68 | -4.56 | -5.02 | -5.39 | -5.03 | -7.88 | -7.33 | -7.83 | H3K36A |
| 350 | -5.89 | -7.42 | -8.15 | -7.45 | -6.92 | -5.86 | -8.46 | -7.92 | -7.85 | H3[del 4-20] |
| 351 | -2.31 | -2.58 | -3.39 | -3.49 | -4.37 | -4.43 | -8.1 | -7.1 | -8.02 | H3R2K |
| 352 | -3.54 | -3.99 | -4.76 | -4.81 | -5.06 | -4.61 | -7.57 | -7.48 | -8.08 | H3S87D |
| 353 | -3.33 | -3.5 | -4.16 | -4.88 | -4.85 | -4.7 | -7.66 | -7 | -8.08 | H4I29A |
| 354 | -3.45 | -4.5 | -5.35 | -4.21 | -4.93 | -5 | -8.26 | -7.37 | -8.16 | H3[del 5-8] |
| 355 | -4.33 | -6.51 | -6.78 | -5.32 | -5.4 | -5.18 | -7.66 | -7.97 | -8.17 | H3[del 5-12] |
| 356 | -2.93 | -3.35 | -4.41 | -4.83 | -4.44 | -4.29 | -8.32 | -7.6 | -8.21 | H3R83A |
| 357 | -5.18 | -6.24 | -6.8 | -5.98 | -7.11 | -5.78 | -7.97 | -6.76 | -8.31 | H3L61A |
| 358 | -2.79 | -3.34 | -4.34 | -4.28 | -4.7 | -4.91 | -8.65 | -7.7 | -8.41 | H3T3D |
| 359 | -6.77 | -6.55 | -6.97 | -6 | -7.18 | -6.61 | -8.8 | -7.75 | -8.45 | H4[del 15-18] |
| 360 | -4.06 | -4.71 | -5.99 | -6.01 | -6.29 | -5.54 | -8.41 | -8.36 | -8.51 | H4T80A |
| 361 | -3.8 | -4.15 | -4.51 | -4.59 | -5.1 | -5.19 | -8.7 | -7.1 | -8.55 | H3Q85A |
| 362 | -4.2 | -4.47 | -5.22 | -4.7 | -5.39 | -5.53 | -7.97 | -7.66 | -8.58 | H3F78A |
| 363 | -3.76 | -4.41 | -5.37 | -6.01 | -5.43 | -4.76 | -7.75 | -7.56 | -8.59 | H3A110S |
| 364 | -4.24 | -4.76 | -5.69 | -5.91 | -6.07 | -5.57 | -7.96 | -7.81 | -8.64 | H3V71A |
| 365 | -4.21 | -4.55 | -5.31 | -5.08 | -4.79 | -5.16 | -7.72 | -7.48 | -8.71 | H3K4R |
| 366 | -6.84 | -8.78 | -9.05 | -6.7 | -7.77 | -6.74 | -9.01 | -9.45 | -8.74 | H3[del 5-24] |
| 367 | -2.6 | -3.32 | -4.58 | -3.86 | -4.53 | -4.99 | -8.22 | -7.53 | -8.75 | H3T3A |
| 368 | -3.19 | -3.45 | -4.31 | -3.94 | -5.09 | -5.23 | -9.06 | -7.76 | -8.78 | H3L100A |
| 369 | -4.14 | -4.02 | -4.55 | -4.93 | -5.32 | -5.25 | -8.5 | -7.91 | -8.81 | H4A89S |
| 370 | -6.92 | -8.54 | -9.64 | -7.68 | -7.63 | -6.92 | -9.04 | -9.77 | -8.84 | H3[del 17-32] |
| 371 | -7.04 | -8.43 | -9.61 | -7.51 | -8.72 | -6.68 | -9.05 | -8.47 | -8.96 | H3[del 13-28] |
| 372 | -4.1 | -4.96 | -5.65 | -5.97 | -5.87 | -5.32 | -8.03 | -7.91 | -9 | H3L92A |
| 373 | -5.41 | -7.51 |  |  | -6.8 | -7.47 |  | -6.26 | -9 | H3K122A |
| 374 | -3.71 | -4.04 | -4.68 | -5.04 | -5.3 | -5.11 | -8.02 | -7.55 | -9.13 | H3I74A |
| 375 | -7.17 | -8.59 | -9.08 | -8.37 | -7.36 | -7.24 | -8.74 | -8.17 | -9.22 | H3K122Q |
| 376 | -3.14 | -3.91 | -4.74 | -3.96 | -5.64 | -5.99 | -9.03 | -8.19 | -9.27 | H3Q5A |
| 377 | -3.47 | -4.05 | -5.06 | -5.45 | -4.81 | -5.21 | -8.24 | -8.46 | -9.32 | H3R2A |
| 378 | -4.12 | -4.25 | -5.11 | -6.2 | -4.93 | -5.17 | -7.57 | -8.36 | -9.32 | H3K4Q |
| 379 | -3.28 | -3.53 | -4.4 | -3.7 | -5.32 | -5.41 | -9.07 | -7.64 | -9.36 | H3S86D |
| 380 | -2.85 | -3.03 | -3.42 | -3.72 | -4.29 | -4.69 | -7.94 | -7.97 | -9.46 | H4I66A |
| 381 | -6.55 | -7.5 | -8.48 | -8.41 | -8.17 | -7.34 | -9.69 | -9.47 | -9.53 | H3V117A |
| 382 | -6.86 | -8.35 | -9.58 | -7.93 | -10.09 | -7.68 | -10.15 | -9.55 | -9.71 | H3[del 21-36] |
| 383 | -5.8 | -6.15 | -7.8 | -6.84 | -8.16 | -6.89 | -10.71 | -10.07 | -9.81 | H3K56Q |
| 384 | -5.62 | -5.33 | -5.22 | -4.39 | -5.84 | -5.74 | -7.88 | -8.73 | -9.87 | H3R69K |
| 385 | -6.36 | -6.13 | -5.95 | -4.15 | -7.4 | -7.44 | -10.01 | -9.03 | -9.94 | H4V81A |
| 386 | -3.71 | -4.06 | -5.13 | -5.31 | -4.78 | -4.94 | -8.88 | -7.92 | -9.95 | H3K4A |
| 387 | -4.21 | -4.69 | -5.89 | -6.2 | -5.65 | -5.74 | -8.96 | -9.13 | -10.07 | H3[del 1-4] |
| 388 | -5.36 | -5.27 | -5.61 | -4.7 | -6.79 | -6.76 | -9.98 | -9.03 | -10.17 | H4R3K |
| 389 | -4.36 | -4.55 | -5.43 | -4.22 | -7.09 | -8.46 |  | -9.88 | -10.21 | H3S95D |
| 390 | -5.17 | -5.17 | -5.56 | -5.03 | -6.78 | -6.96 | -10.18 | -9.46 | -10.23 | H4L37A |
| 391 | -6.34 | -4.92 | -5.36 | -4.35 | -6.69 | -7.03 | -11.22 | -9.06 | -10.24 | H4R78K |
| 392 | -8.37 | -10.47 | -10.99 | -9.92 | -9.02 | -8.85 | -11.04 | -11.22 | -10.38 | H3[del 13-32] |
| 393 | -3.95 | -4.44 | -5.57 | -4.64 | -6.7 | -7.31 | -10.16 | -9.13 | -10.5 | H3Q5E |
| 394 | -6.38 | -7.78 | -9.13 | -5.98 | -9.11 | -7.91 | -12.09 | -10.88 | -10.62 | H3[del 9-20] |
| 395 | -4.8 | -6.2 | -7.49 | -6.29 | -6.29 | -6.42 | -9.93 | -9.21 | -10.68 | H3[del 1-12] |
| 396 | -8.8 | -8.32 | -9.1 |  | -9.19 | -7.7 | -11.47 | -9.07 | -10.81 | H3K56A |
| 397 | -5.97 | -6.79 | -6.73 | -6.35 | -7.36 | -7.45 | -9.05 | -9.91 | -10.97 | H4L97A |
| 398 | -4.93 | -4.52 | -5.21 | -5.3 | -5.75 | -6.15 | -9.28 | -9.23 | -11.43 | H4V86A |
| 399 | -5.75 | -5.54 | -5.7 | -4.67 | -6.8 | -6.82 | -9.69 | -9.69 | -11.45 | H3K18Q |
| 400 | -6.38 | -7.18 | -8.28 | -6.94 | -8.71 | -8.8 | -11.57 | -10.36 | -11.91 | H4D85A |
| 401 | -7.21 | -8.47 | -9.83 | -6.69 | -8.84 | -8.69 | -11.2 | -11.8 | -11.96 | H3[del 13-24] |
| 402 | -6.96 | -6.68 | -7.17 | -6.34 | -7.73 | -7.79 | -11.8 | -11.12 | -12.4 | H4[del 1-4] |
| 403 | -7.23 | -7.2 | -7.21 | -6.53 | -9.86 | -11.41 |  | -13.2 | -12.94 | H3Q68A |
| 404 | -9.87 |  |  | -12.01 | -11.85 | -10.94 |  |  | -13.46 | H3[del 5-20] |
| 405 | -7.35 | -7.5 | -7.35 | -6.67 | -9.38 | -10.17 | -12.23 | -12.09 | -13.83 | H3F104A |
| 406 | -8.37 | -9.03 | -9.09 | -7.04 | -10.05 | -10.5 | -12.91 | -13.1 | -13.84 | H3K4,9,14,18R |
| 407 | -8.51 | -8.1 | -8.13 | -7.1 | -11.36 | -11.45 |  | -11.65 | -13.98 | H3L60A |
| 408 | -6.45 | -6.53 | -7.3 | -6.81 | -11.16 | -10.99 | -12.76 | -14.36 | -14.1 | H3[del 4-15] |
