# Supplementary material for: Distinct structural groups of histone H3 and H4 residues have divergent effects on chronological lifespan in Saccharomyces cerevisiae
Source: PLoS One. 2022 May 27;17(5):e0268760. doi: 10.1371/journal.pone.0268760 (PMC9140238; doi:10.1371/journal.pone.0268760)
Supplement: S2 Table — The number of transcription factor binding sites present within assigned Sir3 peaks was determined and the statistical significance of this number p(k) calculated from a Poisson distribution, e-.λkk!, where k is the number of occurrences per unit length in a Sir3 peak, and λ is equal to the number of genomic occurrences of the site per unit length. (DOCX) [file pone.0268760.s005.docx]

**S2 Table. Significance of transcription factor binding sites present in redistributed Sir3 peaks.** The number of transcription factor binding sites present within assigned Sir3 peaks was determined and the statistical significance of this number *p*(*k*) calculated from a Poisson distribution, $e^{-}.\frac{\lambda^{k}}{k!}$, where k is the number of occurrences per unit length in a Sir3 peak, and λ is equal to the number of genomic occurrences of the site per unit length.

| **Factor** | **Peaks** | **Total peaks** | **Total genome** | **Poisson pmf** | **Description** |
| --- | --- | --- | --- | --- | --- |
| **H4K16Q** | | | | | |
| IME1 | 56 | 146 | 9340 | 1.94E-39 | Inducer of MEiosis |
| PDR1 | 53 | 166 | 14443 | 8.29E-29 | Pleiotropic Drug Resistance |
| STP2 | 54 | 75 | 4832 | 3.14E-21 | Protein with similarity to Stp1p |
| PUT3 | 36 | 116 | 10590 | 1.00E-19 | Proline UTilization |
| SFL1 | 33 | 66 | 36881 | 3.24E-16 | Suppressor gene for FLocculation |
| ZMS1 | 56 | 131 | 14366 | 8.96E-16 | Zinc-finger protein |
| CAD1 | 40 | 102 | 45365 | 5.40E-13 | CADmium resistance |
| NHP10 | 41 | 71 | 6479 | 9.36E-13 | Non-Histone Protein |
| TBS1 | 21 | 81 | 9241 | 1.50E-09 | ThiaBendazole Sensitive |
| OAF1 | 54 | 154 | 22341 | 3.53E-09 | Oleate-Activated transcription Factor |
| STP4 | 52 | 142 | 20357 | 8.30E-09 | Protein with similarity to Stp1p |
| NSI1 | 46 | 71 | 8375 | 3.15E-08 | NTS1 SIlencing protein 1 |
| CIN5 | 53 | 133 | 47599 | 7.24E-08 | Chromosome INstability |
| ABF1 | 67 | 141 | 21284 | 8.58E-08 | ARS-Binding Factor 1 |
| PDR3 | 16 | 34 | 3256 | 5.73E-07 | Pleiotropic Drug Resistance |
| STP3 | 47 | 113 | 17859 | 9.15E-06 | Protein with similarity to Stp1p |
| SPT15 | 24 | 73 | 25001 | 1.98E-04 | TBP basal factor |
| SWI4 | 55 | 133 | 23937 | 3.46E-04 | SWItching deficient |
| MGA1 | 37 | 67 | 22625 | 4.87E-04 |  |
| STB3 | 29 | 48 | 17244 | 6.15E-04 | Sin Three Binding protein |
| PDR8 | 58 | 139 | 25911 | 7.61E-04 | Pleiotropic Drug Resistance |
| **H4H18A** | | | | | |
| IME1 | 30 | 98 | 9340 | 5.20E-22 | Inducer of MEiosis |
| PDR1 | 33 | 116 | 14443 | 1.79E-16 | Pleiotropic Drug Resistance |
| PUT3 | 21 | 87 | 10590 | 2.75E-13 | Proline UTilization |
| SFL1 | 22 | 54 | 36881 | 3.83E-13 | Suppressor gene for FLocculation |
| UGA3 | 31 | 118 | 16818 | 5.86E-13 | Utilization of GAba |
| REB1 | 39 | 131 | 19424 | 5.87E-13 | RNA polymerase I Enhancer Binding protein |
| CAD1 | 22 | 80 | 45365 | 1.84E-11 | CADmium resistance |
| STP2 | 32 | 47 | 4832 | 1.71E-10 | Protein with similarity to Stp1p |
| OAF1 | 37 | 131 | 22341 | 2.83E-09 | Oleate-Activated transcription Factor |
| NHP10 | 26 | 54 | 6479 | 3.74E-09 | Non-Histone Protein |
| ZMS1 | 33 | 91 | 14366 | 1.04E-08 | ReSpiration Factor |
| RPH1 | 39 | 137 | 25055 | 5.19E-08 | Regulator of PHR1 |
| STP4 | 34 | 116 | 20357 | 6.45E-08 | Protein with similarity to Stp1p |
| MET31 | 37 | 145 | 27447 | 1.36E-07 | METhionine requiring |
| NSI1 | 31 | 58 | 8375 | 2.53E-07 | NTS1 SIlencing protein 1 |
| GAL4 | 37 | 122 | 22321 | 2.87E-07 | GALactose metabolism |
| CIN5 | 38 | 110 | 47599 | 3.09E-06 | Chromosome INstability |
| SPT15 | 16 | 48 | 25001 | 4.01E-06 | SuPpressor of Ty insertions |
| TBS1 | 14 | 56 | 9241 | 1.59E-05 | ThiaBendazole Sensitive |
| STP3 | 30 | 93 | 17859 | 1.77E-05 | Protein with similarity to Stp1p |
| RAP1 | 36 | 120 | 24608 | 2.62E-05 | Repressor/Activator site binding Protein |
| ABF1 | 38 | 96 | 21284 | 0.00109 | ARS-Binding Factor 1 |
