# Supplementary material for: Distinct structural groups of histone H3 and H4 residues have divergent effects on chronological lifespan in Saccharomyces cerevisiae
Source: PLoS One. 2022 May 27;17(5):e0268760. doi: 10.1371/journal.pone.0268760 (PMC9140238; doi:10.1371/journal.pone.0268760)
Supplement: S3 Table — (DOCX) [file pone.0268760.s006.docx]

**S3 Table. Annotated genes that are associated with elevated genomic Sir3 levels in WT, H4K16Q, H4H18A and H3E50A yeast strains.**

| **Strain(s) in group** | **Number genes shared** | **Genes shared** |
| --- | --- | --- |
| **E50 H18 K16 WT** | 2 | PHO4 QCR6 |
| **E50 H18 K16** | 1 | YIL055C |
| **K16 WT** | 3 | YFR018C UTR5 IGD1 |
| **H18 K16** | 5 | MAM3 YLR227W-A SND1 YLR227W-B YEL008C-A |
| **E50 K16** | 2 | CMC4 PUT4 |
| **WT** | 4 | RPL13B VBA3 YCL068C HMRA2 |
| **K16** | 21 | GDH1 YGR038C-B GMC1 PRB1 NAT4 LEU4 SYG1 ANP1 RRN5 SCS3 YJL027C MDJ1 YFL015C PAC11 CUE4 PSP1 RSM7 YGR038C-A RIB3 RAX1 YKL097C |
| **H18** | 8 | YGR027W-B YGR027W-A SHR5 MAE1 MDY2 AGP1 YGR050C HTZ1 |
| **E50** | 5 | YCR006C OYE3 YJL133C-A ESL2 CIT2 |
