# Supplementary material for: Distinct structural groups of histone H3 and H4 residues have divergent effects on chronological lifespan in Saccharomyces cerevisiae
Source: PLoS One. 2022 May 27;17(5):e0268760. doi: 10.1371/journal.pone.0268760 (PMC9140238; doi:10.1371/journal.pone.0268760)
Supplement: S4 Table — The reported probability value is calculated as the probability mass function of a hypergeometric distribution using the values in the Enrichment column, and the q-value is the Benjamini–Hochberg corrected p-value. (DOCX) [file pone.0268760.s007.docx]

**S4 Table. GO biological function categories that show significant enrichment for genes that are induced (adjusted p<0.05) in the H4K16Q, H4H18A and H3E50A mutant strains compared to the WT strain.** The reported probability value is calculated as the probability mass function of a hypergeometric distribution using the values in the Enrichment column, and the q-value is the Benjamini–Hochberg corrected p-value.

| **GO biological function** | **Probability (p)** | **FDR q-value** | **Enrichment (N, K, n, k)** |
| --- | --- | --- | --- |
| **H4K16Q mutant** | | | |
| **Cytoplasmic translation** | 5.68E-59 | 2.99E-55 | 3.75 (5768,132,1376,118) |
| **Organonitrogen compound biosynthetic process** | 1.08E-54 | 2.85E-51 | 2.02 (5768,736,1376,355) |
| **Ribonucleoprotein complex biogenesis** | 1.57E-49 | 2.75E-46 | 2.93 (5768,222,1376,155) |
| **Ribosome biogenesis** | 3.76E-45 | 4.94E-42 | 2.97 (5768,195,1376,138) |
| **Cellular component biogenesis** | 1.91E-44 | 2.01E-41 | 2.72 (5768,247,1376,160) |
| **ncRNA metabolic process** | 2.40E-44 | 2.10E-41 | 2.17 (5768,482,1376,250) |
| **rRNA processing** | 6.01E-44 | 4.52E-41 | 2.66 (5768,258,1376,164) |
| **ncRNA processing** | 8.06E-42 | 5.30E-39 | 2.31 (5768,375,1376,207) |
| **Amide biosynthetic process** | 1.55E-38 | 9.05E-36 | 2.27 (5768,370,1376,200) |
| **rRNA metabolic process** | 6.73E-38 | 3.54E-35 | 2.41 (5768,301,1376,173) |
| **H4H18A mutant** | | | |
| **Organonitrogen compound biosynthetic process** | 7.38E-79 | 3.88E-75 | 2.69 (5768,736,899,309) |
| **Cytoplasmic translation** | 4.60E-78 | 1.21E-74 | 5.64 (5768,132,899,116) |
| **Peptide biosynthetic process** | 1.78E-58 | 3.12E-55 | 3.40 (5768,321,899,170) |
| **Translation** | 2.08E-58 | 2.73E-55 | 3.41 (5768,318,899,169) |
| **Amide biosynthetic process** | 4.00E-57 | 4.21E-54 | 3.17 (5768,370,899,183) |
| **Peptide metabolic process** | 1.67E-52 | 1.46E-49 | 3.14 (5768,352,899,172) |
| **Cellular biosynthetic process** | 1.61E-50 | 1.21E-47 | 1.88 (5768,1346,899,394) |
| **Cellular amide metabolic process** | 3.24E-50 | 2.13E-47 | 2.83 (5768,435,899,192) |
| **Organic substance biosynthetic process** | 3.55E-50 | 2.08E-47 | 1.86 (5768,1386,899,401) |
| **Biosynthetic process** | 3.78E-50 | 1.99E-47 | 1.85 (5768,1402,899,404) |
| **H3E50A mutant** | | | |
| **Oxidation-reduction process** | 2.30E-19 | 1.21E-15 | 1.93 (5768,410,1136,156) |
| **Generation of precursor metabolites and energy** | 2.07E-16 | 5.46E-13 | 2.46 (5768,157,1136,76) |
| **Antibiotic metabolic process** | 1.06E-15 | 1.86E-12 | 3.33 (5768,64,1136,42) |
| **Energy derivation by oxidation of organic compounds** | 1.70E-15 | 2.23E-12 | 2.82 (5768,99,1136,55) |
| **Tricarboxylic acid cycle** | 9.40E-14 | 9.89E-11 | 4.23 (5768,30,1136,25) |
| **Citrate metabolic process** | 9.40E-14 | 8.24E-11 | 4.23 (5768,30,1136,25) |
| **Electron transport chain** | 3.17E-13 | 2.38E-10 | 3.26 (5768,56,1136,36) |
| **Respiratory electron transport chain** | 3.61E-13 | 2.37E-10 | 4.00 (5768,33,1136,26) |
| **Tricarboxylic acid metabolic process** | 3.95E-13 | 2.31E-10 | 4.09 (5768,31,1136,25) |
| **Cellular respiration** | 3.13E-11 | 1.65E-08 | 3.03 (5768,57,1136,34) |
