# Supplementary material for: Distinct structural groups of histone H3 and H4 residues have divergent effects on chronological lifespan in Saccharomyces cerevisiae
Source: PLoS One. 2022 May 27;17(5):e0268760. doi: 10.1371/journal.pone.0268760 (PMC9140238; doi:10.1371/journal.pone.0268760)
Supplement: S5 Table — S. cerevisiae KEGG pathways were identified with KEGG Mapper1, and are arranged by descending mapping frequency. (DOCX) [file pone.0268760.s008.docx]

**S5 Table. KEGG pathways associated with genes that are significantly upregulated (adjusted p<0.05) in the H4K16Q, H4H18A and H3E50A mutant compared to the WT strain***. S. cerevisiae* KEGG pathways were identified with KEGG Mapper^1^, and are arranged by descending mapping frequency.

| **Pathway** | **Description** | **Number of genes** |
| --- | --- | --- |
| **H4K16Q** | | |
| **sce01100** | Metabolic pathways | 263 |
| **sce01110** | Biosynthesis of secondary metabolites | 137 |
| **sce03010** | Ribosome | 136 |
| **sce01130** | Biosynthesis of antibiotics | 118 |
| **sce01230** | Biosynthesis of amino acids | 86 |
| **sce01200** | Carbon metabolism | 50 |
| **sce03008** | Ribosome biogenesis in eukaryotes | 47 |
| **sce03013** | RNA transport | 44 |
| **sce00230** | Purine metabolism | 29 |
| **sce01210** | 2-Oxocarboxylic acid metabolism | 26 |
| **H4H18A** | | |
| **sce01100** | Metabolic pathways | 202 |
| **sce03010** | Ribosome | 131 |
| **sce01110** | Biosynthesis of secondary metabolites | 115 |
| **sce01130** | Biosynthesis of antibiotics | 98 |
| **sce01230** | Biosynthesis of amino acids | 69 |
| **sce01200** | Carbon metabolism | 35 |
| **sce00230** | Purine metabolism | 28 |
| **sce03013** | RNA transport | 27 |
| **sce00010** | Glycolysis / Gluconeogenesis | 23 |
| **sce03008** | Ribosome biogenesis in eukaryotes | 22 |
| **H3E50A** | | |
| **sce01100** | Metabolic pathways | 195 |
| **sce01110** | Biosynthesis of secondary metabolites | 78 |
| **sce01130** | Biosynthesis of antibiotics | 60 |
| **sce01200** | Carbon metabolism | 49 |
| **sce00190** | Oxidative phosphorylation | 38 |
| **sce00620** | Pyruvate metabolism | 27 |
| **sce00020** | Citrate cycle (TCA cycle) | 26 |
| **sce04138** | Autophagy | 24 |
| **sce01230** | Biosynthesis of amino acids | 20 |
| **sce04146** | Peroxisome | 20 |
| ^1^Kanehisa, M., Goto, S., Sato, Y., Furumichi, M. & Tanabe, M. KEGG for integration and interpretation of large-scale molecular data sets. Nucleic Acids Res. 40, D109-14 (2012). | | |
