# Supplementary material for: Distinct structural groups of histone H3 and H4 residues have divergent effects on chronological lifespan in Saccharomyces cerevisiae
Source: PLoS One. 2022 May 27;17(5):e0268760. doi: 10.1371/journal.pone.0268760 (PMC9140238; doi:10.1371/journal.pone.0268760)
Supplement: S6 Table — The reported probability value is calculated as the probability mass function of a hypergeometric distribution using the values in the Enrichment column, and the q-value is the Benjamini–Hochberg corrected p-value. (DOCX) [file pone.0268760.s009.docx]

**S6 Table. GO biological function categories that show significant enrichment for genes that are repressed (adjusted p<0.05) in the H4K16Q, H4H18A and H3E50A strains compared to the WT strain.** The reported probability value is calculated as the probability mass function of a hypergeometric distribution using the values in the Enrichment column, and the q-value is the Benjamini–Hochberg corrected p-value.

| **GO biological function** | **Probability (p)** | **FDR q-value** | **Enrichment (N, K, n, k)** |
| --- | --- | --- | --- |
| **H4K16Q** | | | |
| **DNA integration** | 1.41E-14 | 7.40E-11 | 4.02 (5768,49,938,32) |
| **DNA recombination** | 1.55E-14 | 4.08E-11 | 2.31 (5768,213,938,80) |
| **Reproductive process** | 5.97E-11 | 1.05E-07 | 1.76 (5768,401,938,115) |
| **Meiotic cell cycle** | 7.45E-11 | 9.80E-08 | 2.41 (5768,135,938,53) |
| **RNA-dependent DNA biosynthetic process** | 9.10E-11 | 9.57E-08 | 3.07 (5768,68,938,34) |
| **Meiotic cell cycle process** | 6.52E-09 | 5.71E-06 | 1.89 (5768,244,938,75) |
| **Transposition** | 2.56E-07 | 1.93E-04 | 2.30 (5768,99,938,37) |
| **DNA biosynthetic process** | 3.38E-07 | 2.22E-04 | 2.31 (5768,96,938,36) |
| **Transposition, RNA-mediated** | 4.45E-07 | 2.60E-04 | 2.31 (5768,93,938,35) |
| **Mitochondrial respiratory chain complex assembly** | 2.55E-06 | 1.34E-03 | 2.81 (5768,46,938,21) |
| **H4H18A** | | | |
| **DNA integration** | 8.96E-13 | 4.71E-09 | 3.08 (5768,49,1338,35) |
| **DNA recombination** | 1.78E-12 | 4.68E-09 | 1.92 (5768,213,1338,95) |
| **RNA-dependent DNA biosynthetic process** | 8.93E-12 | 1.57E-08 | 2.66 (5768,68,1338,42) |
| **DNA biosynthetic process** | 2.04E-09 | 2.68E-06 | 2.20 (5768,96,1338,49) |
| **Cell cycle process** | 1.23E-07 | 1.29E-04 | 1.39 (5768,571,1338,184) |
| **DNA metabolic process** | 1.39E-07 | 1.22E-04 | 1.43 (5768,480,1338,159) |
| **Meiotic cell cycle** | 1.36E-06 | 1.02E-03 | 1.79 (5768,135,1338,56) |
| **Cell cycle** | 1.21E-05 | 7.95E-03 | 1.38 (5768,408,1338,131) |
| **Meiotic cell cycle process** | 1.69E-05 | 9.90E-03 | 1.50 (5768,244,1338,85) |
| **Reproductive process** | 2.10E-05 | 1.11E-02 | 1.38 (5768,401,1338,128) |
| **H3E50A** | | | |
| **Ribonucleoprotein complex biogenesis** | 1.67E-47 | 8.81E-44 | 2.93 (5768,222,1341,151) |
| **Cellular component biogenesis** | 6.97E-47 | 1.83E-43 | 2.80 (5768,247,1341,161) |
| **Ribosome biogenesis** | 6.73E-43 | 1.18E-39 | 2.96 (5768,195,1341,134) |
| **Cytoplasmic translation** | 3.21E-40 | 4.22E-37 | 3.32 (5768,132,1341,102) |
| **rRNA processing** | 2.00E-36 | 2.10E-33 | 2.53 (5768,258,1341,152) |
| **rRNA metabolic process** | 1.68E-33 | 1.48E-30 | 2.34 (5768,301,1341,164) |
| **ncRNA metabolic process** | 6.41E-29 | 4.81E-26 | 1.95 (5768,482,1341,218) |
| **ncRNA processing** | 1.11E-28 | 7.30E-26 | 2.09 (5768,375,1341,182) |
| **Maturation of SSU-rRNA** | 8.15E-22 | 4.76E-19 | 3.17 (5768,80,1341,59) |
| **maturation of SSU-rRNA from tricistronic rRNA transcript (SSU-rRNA, 5.8S rRNA, LSU-rRNA)** | 4.24E-21 | 2.23E-18 | 3.34 (5768,67,1341,52) |
