# Supplementary material for: Distinct structural groups of histone H3 and H4 residues have divergent effects on chronological lifespan in Saccharomyces cerevisiae
Source: PLoS One. 2022 May 27;17(5):e0268760. doi: 10.1371/journal.pone.0268760 (PMC9140238; doi:10.1371/journal.pone.0268760)
Supplement: S7 Table — S. cerevisiae KEGG pathways were identified with KEGG Mapper, and are arranged by descending mapping frequency. (DOCX) [file pone.0268760.s010.docx]

**S7 Table. KEGG pathways associated with genes that are repressed (adjusted p<0.05) in the H4K16Q, H4H18A and H3E50A mutant compared to the WT strain***. S. cerevisiae* KEGG pathways were identified with KEGG Mapper, and are arranged by descending mapping frequency.

| **Pathway** | **Description** | **Number of genes (of 844)** |
| --- | --- | --- |
| **H4K16Q** | | |
| **sce01100** | Metabolic pathways | 73 |
| **sce04113** | Meiosis | 37 |
| **sce01110** | Biosynthesis of secondary metabolites | 29 |
| **sce04111** | Cell cycle | 27 |
| **sce04011** | MAPK signaling pathway | 22 |
| **sce01130** | Biosynthesis of antibiotics | 20 |
| **sce04138** | Autophagy | 14 |
| **sce01200** | Carbon metabolism | 13 |
| **sce03010** | Ribosome | 11 |
| **sce04144** | Endocytosis | 10 |
| **H4H18A** | | |
| **sce01100** | Metabolic pathways | 108 |
| **sce04113** | Meiosis | 39 |
| **sce04111** | Cell cycle | 36 |
| **sce01110** | Biosynthesis of secondary metabolites | 33 |
| **sce04011** | MAPK signaling pathway | 28 |
| **sce01130** | Biosynthesis of antibiotics | 25 |
| **sce04138** | Autophagy | 23 |
| **sce03040** | Spliceosome | 19 |
| **sce01200** | Carbon metabolism | 16 |
| **sce04146** | Peroxisome | 15 |
| **H3E50A** | | |
| **sce01100** | Metabolic pathways | 192 |
| **sce03010** | Ribosome | 123 |
| **sce01110** | Biosynthesis of secondary metabolites | 77 |
| **sce01130** | Biosynthesis of antibiotics | 65 |
| **sce01230** | Biosynthesis of amino acids | 41 |
| **sce04111** | Cell cycle | 40 |
| **sce03008** | Ribosome biogenesis in eukaryotes | 39 |
| **sce04113** | Meiosis | 33 |
| **sce04011** | MAPK signaling pathway | 33 |
| **sce00230** | Purine metabolism | 27 |
