# Supplementary material for: Distinct structural groups of histone H3 and H4 residues have divergent effects on chronological lifespan in Saccharomyces cerevisiae
Source: PLoS One. 2022 May 27;17(5):e0268760. doi: 10.1371/journal.pone.0268760 (PMC9140238; doi:10.1371/journal.pone.0268760)
Supplement: S1 Note — (DOCX) [file pone.0268760.s011.docx]

**S1 Note. Analysis of structure and interactions of residues implicated in chronological lifespan.**

**Residues implicated in extension of lifespan**

To understand the impact of mutating the residues at the histone fold fringes on nucleosome structure, we identified the interactions of the wild type residues at each position in the nucleosome. In the H3 C-terminal region of α3, R128 and R131 make H-bonds to H3Y99 and H3D106 in the central region of α2, and H3R131 makes an H-bond to R99 in the Cα helix of H2A (S Fig. 1a). These interactions probably stabilise the position of the C-terminus of H3 in the nucleosome and contribute to the formation of a stable four helix bundle connecting the two H3-H4 dimers. H3K115 is oriented towards a phosphate group in the DNA sugar-phosphate backbone, and H3I112 forms a hydrophobic cluster with H2AQ112 and H2AV114 (S Fig. 1b,c). H3Q76 makes bridging H-bonds to the backbone at H3D81 in L1 and H3R72 in α1, which may stabilize the L1 loop conformation (S Fig. 1d). Whereas substitution of H3Q76 with E extends lifespan, substitution with A reduces lifespan to less than the median of the population (S Table 1). H3R83 is inserted into the minor groove at position SHL ±2.5 (S Fig. 1e). Arginine is inserted into the minor groove at six other positions at SHL ±6.5 (H3R49), ±5.5 (H2AR47) and ±0.5 (H4R45), giving a total of 8 such insertions over the length of the nucleosome DNA duplex [1]. H3P66 at the N-terminus of α1 may contribute to the termination the α1 helix and formation of a coil between the αN and α1 helices of H3 (S Fig. 1f). In the region of the structure where H4 α1 passes over H3 αN, H3Q55 is hydrogen bonded to the H2A C-terminal tail backbone at N110 as well as to R40 in H4 α1 (S Fig. 1g). H4R36 is oriented towards the DNA, and makes a salt bridge with a backbone phosphate (S Fig. 1h). As expected, the charge of the residue is crucial, since whereas the R🠖K substitution extends lifespan, the R🠖A change reduced lifespan to less than the population median (S Table 1). H4R40 makes H-bonds to the backbone at positions in the C-terminus of αN and the coil connecting αN and α1 of H3 (S Fig. 1i). I50 at the N-terminus of H4 α2 is inserted between H3I119 and H3I124, allowing hydrophobic contact, and fixing the position of the N-terminus of H4 α2 to α3 of H3 (S Fig. 1j). H75 in the C-terminus of H4 α2 make hydrophobic contact with L77 in the α2 C-terminus and T93 in the centre of α3 of H2B, as well as H-bonds to R89 at the N-terminus of H2B α3 (S Fig. 1k). These interactions will contribute to the stability of the four helix bundle that binds a H3-H4 dimer to a H2A-H2B dimer. F100 in the short C-terminal tail of H4 makes numerous hydrophobic contacts with A83 and V87 in the α3 of H4, as well as L97 in the C-terminal β-strand. Additional contacts are also seen between H4F100 and S87 and A91 in the N-terminal part of the H3 α2 (S Fig. 1l).

All these interactions, apart from being concentrated at the extremities of the histone fold domains of H3 and H4, involve interactions either with other dimers, interactions between the αN and α1 domains of H3 and H4, or interactions with the DNA duplex. The interactions specifically exclude the extensive interactions between dimer partners and seem enriched for contacts that stabilize the H3-H4 dimer in its frame within the nucleosome structure.

**Residues implicated in reduction of lifespan**

H3F104 interleaves with a cluster of hydrophobic residues composed of H4L37, H4V43 and H3I119 (S Fig. 1n), and is likely to contribute to the stable placement of the C-terminal region of the α2 helix and the L2 loop of H3 relative to the α1 helix and L1 loop of H4. H3L60 in the coiled region, joining the Nα and α1 helices, approaches H3Q93 in the α2 loop, and H3Q68 in α1 approaches α2 H3V89, setting the path of the H3 tail to a trajectory between the DNA gyres (S Fig. 1o). H4D85 is hydrogen bonded to H4R78 and H4T72, possibly stabilizing the tight turn of L2 between α2 and α3. We speculate that the mutations that cause a shortened lifespan contribute to the structural destabilization of the histone octamer and disruption of chromatin structures.

**References for Supplementary Note**

1. Luger, K., M Auml Der, A. W., Richmond, R. K., Sargent, D. F. & Richmond, T. J. Crystal structure of the nucleosome core particle at 2.8Å resolution. Nature 389, 251 (1997).
