# Supplementary material for: Distinct structural groups of histone H3 and H4 residues have divergent effects on chronological lifespan in Saccharomyces cerevisiae
Source: PLoS One. 2022 May 27;17(5):e0268760. doi: 10.1371/journal.pone.0268760 (PMC9140238; doi:10.1371/journal.pone.0268760)
Supplement: Attachment — Submitted filename: Ngubo_Reid_Patterton_Response to Reviewers V3.docx [file pone.0268760.s012.docx]

PONE-D-21-32183

Distinct structural groups of histone H3 and H4 residues have divergent effects on chronological lifespan in Saccharomyces cerevisiae

PLOS ONE

Reviewer #1: This is a thoughtful and complete study analyzing the affect of histone mutations on cell aging and the related physiology and gene expression changes. It is a very well conceived and executed study and I learned a lot from reading it. I have only major comment.

1.These must have been a mistake in uploading the figures. Figure 2 is missing and Figure 4 is presented in its place. This is true in the merged document and the linked file.

*We thank the reviewer for the comments and the correction, this has been rectified.*

Reviewer #2: In this manuscript, Patterton and colleagues have systematically tested the impact of histone point mutants on chronological lifespan (CLS). They identified 4 structural groups of mutants with distinct effects on lifespan. The paper focuses on the residues increasing lifespan. One group of residues showing chronological lifespan expansion (CLE) is involved in the interaction with the yeast heterochromatin factor Sir3. They performed ChIP experiments on representative mutants from which they conclude that mutants belonging to group 1 show a redistribution of Sir3 at secondary sites in the genome. Some of these new Sir3 binding sites are associated with genes previously reported to increase CLS when deleted. This could represent a direct cause to the observed phenotype. Surprisingly, the author performed transcriptome analysis in late log phase, which analysis is not shown in the main figures. Transcript levels of Sir3 bound genes should be shown as a main figure, especially the one that could be responsible for lifespan expansion.

In general the paper si not very well organized and some relevant references are missing (see below).

Specific comments:

1. The literature describing chromatin reorganization in quiescent cells is not cited (e.g. papers from the Tsukiyama and Taddei lab), ChIP against Sir3 in histone point mutants published by the Grunstein lab for exponential growing cells should be cited and compared with the results presented here.

*We thank the reviewer for the suggestions. Accordingly, we have cited the work from the Taddei and Tsukiyama lab in lines 110-111. We are currently unable to compare the Grunstein lab data with our results, the GEO data is in an old BAR format, and we have tried a number of utilities but could not find a script or program that could successfully convert the bar format to wig. We would appreciate it if the reviewer can suggest a script or program that he/she knows work.*

2. The material and methods section is incomplete:
-The authors should explain how cells are grown over 50 days in flasks. How is the evaporation compensated for? Is the volume adjusted on a regular basis? If not, there must be some volume variation, thus affecting cell concentration and potentially biasing the lifespan assay. A detailed procedure is required here.

*Evaporation of the media was minimized* *by using aluminum foil caps as previously described by Hu 2012, Cell Senescence; Barre 2020, Genome Res., shown in line 561-562. Volume adjustment by introducing fresh media would disrupt the steady state conditions and would encourage cells to re-enter the cycle, exiting G0 phase which is important in examining chronological aging.*

-It is not clear whether the ChIP experiments were performed on sorted Quiescent cells same as for the bar-code sequencing (sorted on percoll gradient) or not.

*The fraction of quiescent cells in stationary phase is an order of magnitude less than non-quiescent cells, because of this very big difference we have performed the ChIP experiments in bulk cells. We have addressed this in the new line 607-608. We have attached an image of the percoll gradient at exponential and stationary phase showing the difference as an illustration to the editor.*

3. Figure 2: Some strains show an increase of CFU during the first days, meaning that they did not reach saturation at day 2. This is a problem as we may not look at an increased lifespan but just a delay of the culture. Once they have reached a maximum, CFUs decrease faster than the wild type.

*The initial increase of cells during the first days can be attributed to the mother cells undergoing one more asymmetric cell division after exponential phase (Werner-Washburne 1993, Microbiol Rev; Herman 2002 Curr Opin Microbiol; Miles 2020, Yeast Extract). The initial increase in chronologically aged yeast is observed by many other groups in the field (Picazo 2015, Plos ONE; Wierman 2017, MCB; Ogawa 2016, PNAS). Since the cells are gown at equivalent initial concentration the initial increase is inferred to fitness of the strain in growth limiting stationary phase culture conditions. We use the definition of chronological lifespan as described in the field by Kennedy, Longo, Werner-Washburne, Guarente, Sinclair, and others that define chronological aging as the length of time a population of yeast cells remains viable in a non-dividing state following nutrient deprivation.*

4. For the mutants showing redistribution of Sir3 on chromatin it would be important to rule out that the observed effect is not due to some pseudo-diploid effect that are observed when the HM loci are derepressed. This would require repeating the lifespan experiments for at least one mutant in a strain deleted for the relevant HM. The authors alluded to this potential caveat in the discussion but ruled it out by citing ref 48, which is about replicative lifespan and not chronological lifespan.

*A thorough search of the literature did not reveal any study that investigated the effect of an E or I element deletion, and expected associated derepression of HMLalpha (or HMRa) on chronological lifespan. We did identify a single study where extension of replicative lifespan was associated with increased HM* ***repression*** *(Kim et al 2017, MBoc). Thus, although we could not specifically identify a study addressing chronological lifespan, all published data that relate lifespan and HM silencing suggest a direct relationship, furthermore, the loss of HM silencing has been showed to be an effect of aging (Kennedy et al.,1997, Sinclair and Guarente, 1997, Smeal et al., 1996). Also, deletion of MATalpha2 causes derepression of HMLalpha. However, this strain is not associated with extension of chronological lifespan in phenotypic survey studies (Laschober et al 2010, Aging Cell; Campos et al 2018, Aging Cell), strongly suggesting that derepression of HM is not causative of chronological lifespan extension. We do not suspect that mating sterility, a phenotype of HM derepression could be a cause of longevity, whereas strong evidence suggest that it is an effect.*

5. Sir3 secondary binding sites have been reported upon Sir3 overexpression (Radman-Livaja et al. 2011; Hocher et al. 2018; Ruault et al, 2021). Are the secondary sites reported here similar? What is the prediction from the model shown in Figure 7?

*There is minimal overlap of the Sir3 secondary binding sites in the histone mutants and the secondary binding sites induced by Sir3 overexpression (Hocher et al. 2018; Ruault et al, 2021), suggesting that the recruitment of Sir3 to the alternative sites that cause lifespan extend does not resemble the same recruitment patterns as in cells with increased concentrations of Sir3. Out of 18 genes in the Sir3 euchromatin sites in the overexpressed Sir3 background strains, only 3 genes overlap with the histone mutants’ secondary site genes, and none in the extended silent domain genes. Therefore, our proposed model demonstrates systematic redistribution of Sir3 to regions that are key for CLE compared to ectopic Sir3 expression. Furthermore, overexpression of Sir3 is not associated with chronological lifespan extension (Butcher et al, 2006 Nature Chemical Biology; Yoshikawa et al 2011, Yeast).*

6. Also, it would have been nice to have the exponential culture conditions as a comparison with the stationary phase (6 days) for the ChIP experiments, at least for the WT strain and histone mutants for which these data sets are available in the literature (see point 1).

*Please see response to point1*

7. I found very surprising that the transcriptome analysis is not shown as a main figure. Transcript levels of the genes associated with new Sir3 binding sites, and previously described to impact lifespan when deleted (GDH1, AGP1, HTZ1 and SHR5 genes) should be shown as a main figure.

*We have shown normalized transcript levels of genes linked with new Sir3 binding sites, and implicated in lifespan extension in new Fig6.*


Minor comments:

Figure 1a: The trace of the log2 levels of the parental WT and median strain are not possible to see.

*We have shown the median strain in the new Fig1a and Fig1e.*

Line 183: maybe it is not the tail which is necessary but just the H3K4 (as stated lane 195-6).

*Other residues outside of the H3K1-4 region also showed decreased lifespan, see Figure 1i, suggesting that the tail is important for normal lifespan, not only a single H3K4 residue.*

Line 227:
-It is not clear why the chip-seq was done at 6 days. It should have been compared to the exponential cultures. Are the Sir3 peaks identified later at 6 days already present in expo? What about the transcriptional status of those genes? When they are affected in late log phase, we would like to know what was their expression levels in expo and at day 6.

*Stationary phase day 6 is day 0 as far as lifespan is concerned, thus, we were interested in the state of chromatin and distribution of Sir3 at the point where we started measuring chronological lifespan.*

*As we have noted in line 352-356, in yeast, there is a well-documented transcriptional shutdown in stationary phase. We appreciate that transcription or repression of certain genes may underpin CLE, however, we were interested in the transcriptome profile that preceded stationary phase, and that may prime cells for later lifespan extension in stationary phase.*

-Also: maybe repeat here that 6 days is stationary phase (not obvious for everybody).

*We have reiterated this accordingly in the new line 233.*

Line 233: Sir3 seems to be enriched in the Y’ element in the H4K16Q strain compared to other? any comments?

*Our quantification of the total signal over the entire Y’ element shows comparable Sir3 enrichment in the H4K16Q strain to the other strains as indicated in Fig3c. The slight difference in enrichment could be strain specific and is non-significant.*

Figure 4: the representation of the statistical data is not clear. Should use other character to differentiate the groups or colors. It is difficult to read it as it is presented now. Since the figures are not heavy, I suggest to plot the count density from the core on a separated plot, in addition to the existing plot to have a better resolution.

*We have presented the data in a standard box plot with multiple t-test comparisons. The p-value symbols are clearly illustrated and explained in the figure legend.*

Figure 5:
-The figure is not really in agreement with the statement that Rap1 levels are not changing in the H18 mutant (it goes from 2 to 3.2 for Rap1, For Sir3, going from 1.2 to 1.35 was significant).

*The reviewer should kindly remember that the statistical t-test analysis does not look at only the means of the samples but it also takes into account the standard deviations and the number of the set size, which all factor in the calculation of statistical significance (p-value) in the changes observed.*

-This is a rather rough analysis. It would be important to show Rap1 profiles over chromosomal regions and to compare Rap1 peaks in the different conditions (expo phase and stationary phase).

*Please see Supplementary Fig. 2a where we showed Rap1 peaks for the different conditions over 15kb chromosome XII chromosomal region.*

Line 298: it is worth mentioning that Abf1 and Rap1 are also found at HM silencers. However, there are many other transcription factors associated with these Sir3 peaks. Only Abf1 and Rap1 are mentioned but they are not the most significant (according to table 2).

*For the purpose of this study, we focused on Abf1 and Rap1 because of their known biological importance in directly interacting with Sir3.*

Supplementary table 1 is not available.

*The Supplementary table 1 has been included.*

Supplementary table 3 : whenever possible, gene’s name should be provided (like in sup2)

*Gene names have been provided where possible. See Supplementary table 3.*

Line 557: replace “performed” by “preformed”

*We thank the reviewer for catching typo, it has been corrected.*
