# Supplementary material for: Distinct structural groups of histone H3 and H4 residues have divergent effects on chronological lifespan in Saccharomyces cerevisiae
Source: PLoS One. 2022 May 27;17(5):e0268760. doi: 10.1371/journal.pone.0268760 (PMC9140238; doi:10.1371/journal.pone.0268760)
Supplement: Attachment — Submitted filename: Ngubo_Reid_Patterton_Response to Reviewers.docx [file pone.0268760.s013.docx]

Reviewers' comments:

Reviewer's Responses to Questions

Reviewer #2: This new version of the manuscript is very similar to the previous one and most of my comments were not considered. This is clearly illustrated by the track-change version of the manuscript in which only few changes can be seen. Yet, I have some comments on these changes:
p6: "In addition, quiescence has been shown to be essential in determining the chromatin dynamics involved in longevity."
This sentence (added in response to my first comment) does not fil well in this section, it is difficult to understand why this information is provided at this stage and not in the introduction.

*We have moved the sentence to the introduction. See line 74-76.*

p29: The ChIP experiments were performed on the bulk cells because the fraction of quiescent cells in stationary phase is significantly less than the non-quiescent cells."
Is this correct? If true, the ChIP is thus performed (mainly)) on non-quiescent cells? Is this proportion the same for all strains?

*We thank the reviewer for seeking this clarification, indeed, we have made an error in the mentioned statement. For the long living cells, the fraction of* ***non-quiescent cells*** *at the onset of stationary phase in day 6 (where we performed our Sir3 ChIP experiments) is significantly less than the* ***quiescent cells****,* *however, at day 55 as the* ***quiescent cells*** *age and accumulate damage the* ***non-quiescent cells*** *increase.* *Accordingly, the ChIP experiments were performed mainly on* ***quiescent cells*** *at day6 in stationary phase. This proportion difference is the same for five of the tested top10 strains. We have attached an image (Appendix 1) with representative strains for illustration at day2, day6 and day55 for the mutant strains, and day2, day7 and day34 for WT.*

Figure 2 has been corrected in response to reviewer’s comment. Indeed, this figure was missing in the first version. It would be important to show the variability of these growth curves. Although the main text and the figure legend specify that growth curves were done in biological replicate, there is no error bars in the plot.

*New Figure 2* *with error bars showing the standard deviation has been included.*

Figure 6 has been completed to answer one of my comments (point 7). Panel b and c have been added to show the normalized transcript levels of genes linked with new Sir3 binding sites and implicated in lifespan extension.
First, here again there are no error bars. Second, from this figure one can clearly see that the GDH1 gene is not repressed in the H4K16 mutant, contrary to what is written l332-333
“The GDH1 glutamate dehydrogenase gene is repressed in the H4K16 mutant strain and is associated with a CLE phenotype in a null strain”; the MAM3 and YIL055C that are repressed in the H4K16 mutant (according to Figure 6), but these genes don’t induce CLE when deleted (as stated l314 and L316 respectively). Therefore, there is no ‘simple, causative link between Sir3 redistribution and lifespan expansion” for the H4K16 mutant (in contradiction with l338).
Regarding the H4H18 mutant, only SHR5 is bound by Sir3, repressed, and associated with CLE.

*We thank the reviewer for correctly pointing this out. The bar graphs in (Figure 6b, c) were made from the FPKM normalization of the count data which is not suitable for showing transcriptomic differences across samples. We have now showed a new (Figure 6c, d) with the bar graph of log2 fold change from differential expression analysis using the DESeq2 package which is suitable for between-samples comparison. New Figure 6c shows significant repression of GDH1 gene in the H4K16 mutant. The MAM3 and YIL055C genes are not repressed in both H4K16 and H4H18 mutant strains (Figure 6c, d). Additionally, AGP1 and HTZ1 genes are repressed in the H4H18 mutant strain consistent with the original text.*

*Regarding the error bars, DESeq2 employs the Wald statistical test* *where the estimated standard error of a log2 fold change is tested. The Wald statistical test* *use the biological replicates we have performed. Moreover, we attach an image (Appendix 2) illustrating the dispersion of variance and the density distribution of the counts, suggesting that the datasets are appropriate to detect small degrees of variability between the levels of expression in different sets.*

Comments on the non-answered comments:
Among the 7 main points raised in my review only 2 have been completely addressed (point 2 and 7)

Point 1 and 6: Sir3 ChIP seq data are readily accessible for WT cells in exponential phase and it would be important to compare these with Sir3 ChIP on stationary phase cells. As for the data from the Grunstein lab on histone mutants, it is always possible to re-analyze the raw data using the software and parameters described in the original paper, and export the files in the appropriate format.

*We thank the reviewer for the suggestion to compare our data with publicly available exponential phase Sir3 ChIP-seq data. Indeed, as we expected the Sir3 occupancy at HM loci is enhanced in the exponential and stationary phase WT compared to the H4K16 and H4H18 mutant strains, accordingly, upstream of SND1 there is reduction in Sir3 occupancy in the exponential and stationary phase WT compared to the H4K16 and H4H18 mutant strains. See new (Figure 3b, d) and Figure 6b.*

*Concerning the Grunstein lab data on histone mutants, as we have mentioned in our previous response, the Affymetrix Tiling Array Software raw data outputs binary files (.bar and .cel) which we have tried different scripts to export to bigwig files, with no success. We even reached out to the first author who told us they never exported the bar files to bigwig files.*

Point 3: I don’t understand how the authors can say that they “define chronological
aging as the length of time a population of yeast cells remains viable in a non-dividing state following nutrient deprivation », while their interpret “The initial increase of cells during the first days […] to the mother cells undergoing one more asymmetric cell division after exponential phase.

*The reviewer expressed a concern that some of the strains increased after day2 and therefore did not reach saturation, which suggests that the authors are looking at delayed cultures. To reiterate our response, we state that the initial increase of cells observed in our cultures is characteristic of post-diauxic growth where mother cells in the culture undergo one more cell division or slow growth into daughter cells that will become quiescent cells (Werner-Washburne 1993, Microbiol Rev; Herman 2002 Curr Opin Microbiol; Miles 2020, Yeast Extract). The survival of the initially increased cells farther into stationary phase is due to the fitness of the long living strains in culture conditions that are not favourable for growth, and not delayed growth as suggested by the reviewer. In stationary phase, glucose is exhausted and the nutrients are limited, deterring growth of unfit strains as seen in the WT and the short living strains, unless the strains are fit to withstand these culture conditions as seen in long living mutant strains.*

Point 4: In their lengthy response to this point, the authors continue to confuse CLS and RLS and do not really answer the main question, which is whether there is a pseudo-diploid effect.

*We apologize to the reviewer for not being clear in our response. We will try to unpack our response again here. The pseudo-diploid effect occurs as a result of derepression of the MATa and MATalpha genes at the HM loci in haploid cells, which has been suggested to cause mating type defect or mating sterility (Dörfel 2017, Yeast). Our argument is that if the pseudo-diploid effect causes sterility, an effect of aging, in RLS we don't suspect that it would cause longevity in CLS. More importantly, the overexpression of MATalpha1 gene in a haploid MATa strain was associated with short lived strains in* ***CLS*** *(Pogoda 2021, Biogerentology), strongly suggesting that derepression of HM loci, or the pseudo-diploid effect is not causative of chronological lifespan extension. We have replaced the RLS reference with the CLS study. See line 470-471.*

Point 5: I found interesting that “There is minimal overlap of the Sir3 secondary binding sites in the histone mutants and the secondary binding sites induced by Sir3 overexpression”. I think this should be mentioned in the manuscript and whether it was predicted by their model or not.

*We are happy to mention this in our discussion. See line 494-499.*
